# Structures and Activity of New Anabaenopeptins Produced by Baltic Sea Cyanobacteria

**DOI:** 10.3390/md14010008

**Published:** 2015-12-30

**Authors:** Lisa Spoof, Agata Błaszczyk, Jussi Meriluoto, Marta Cegłowska, Hanna Mazur-Marzec

**Affiliations:** 1 Biochemistry, Faculty of Science and Engineering, Åbo Akademi University, Tykistökatu 6 A, 20520 Turku, Finland; jussi.meriluoto@abo.fi; 2 Department of Marine Biotechnology, University of Gdańsk, Al. Marszałka Piłsudskiego 46, 81-378 Gdynia, Poland; agata.blaszczyk@ug.edu.pl (A.B.); ma.ceglowska@gmail.com (M.C.); biohm@ug.edu.pl (H.M.-M.)

**Keywords:** Anabaenopeptins, nodulapeptins, Baltic Sea, cyanobacteria, *Nodularia spumigena*, *Aphanizomenon flos-aquae*, *Dolichospermum* spp., carboxypeptidase A, protein phosphatase 1

## Abstract

Anabaenopeptins, bioactive cyclic hexapeptides, were isolated by preparative reversed-phase high performance liquid chromatography from an extract of Baltic Sea cyanobacterial bloom material composed of *Nodularia spumigena* (50%), *Aphanizomenon flos-aquae* (40%) and *Dolichospermum* spp. (10%). Five new anabaenopeptins and nine previously known anabaenopeptins were isolated, and their putative structures were determined by tandem mass spectrometry. The activity of the peptides against carboxypeptidase A and protein phosphatase 1 as well as chymotrypsin, trypsin and thrombin was tested. All anabaenopeptins inhibited carboxypeptidase A (apart from one anabaenopeptin variant) and protein phosphatase 1 with varying potency, but no inhibition against chymotrypsin, trypsin and thrombin was observed.

## 1. Introduction

Marine and freshwater cyanobacteria are one of the most interesting sources of novel biologically active compounds including cytotoxic metabolites, protease inhibitors and antimicrobial agents [1,2,3]. Many secondary metabolites of cyanobacteria are cyclic or linear peptides. Most cyanobacterial peptidic compounds belong to distinct oligopeptide families or classes, such as micropeptins, anabaenopeptins, aeruginosins, spumigins, cyclamides, microginins, microviridins, and the more extensively studied hepatotoxins microcystins and nodularins [4,5].

Anabaenopeptins were named after the cyanobacterium *Anabaena flos-aquae* from which the first anabaenopeptins were isolated as minor compounds accompanying microcystins and the rare neurotoxic alkaloid anatoxin-a(S) [6]. To date, at least 96 anabaenopeptins have been reported in the literature (Supplementary Material Table S1). Whilst these peptides are structurally related, with amino acid substitutions being responsible for the observed diversity, their nomenclature is not fully systematic [4]. Their names often refer to the taxon from which they have been identified or a geographic locality of discovery, complemented with suffices describing the variety. Some anabaenopeptins, especially those identified lately, have the molecular weight as one part of the name, which may be confusing since several anabaenopeptins can have the same molecular weight. Some compounds structurally recognized as anabaenopeptin variants were named in the original papers as oscillamides, isolated from *Planktothrix (Oscillatoria)* [7], nodulapeptins (NPs) from *Nodularia* [8] and ferintoic acids from *Microcystis* [9]. These compounds were discovered concomitantly with anabaenopeptins A to F [10,11]. Anabaenopeptins such as lyngbyaureidamides A and B were isolated from freshwater *Lyngbya* sp. [12] while marine *Lyngbya* sp. has been the source for pompanopeptin B [13], a compound similar to anabaenopeptin J and I originally found in *Aphanizomenon* [14]. The first marine anabaenopeptin-related compounds, keramamides [15], konbamide [16], and mozamides [17] were from the sponge *Theonella* originating from Japan and Mozambique. Brunsvicamides A–C isolated from the cyanobacteria *Tychonema* sp. (order Oscillatoriales) are closely related in structure to the marine sponge-derived anabaenopeptins [18] indicating a cyanobacterial origin [19]. Schizopeptin, isolated from a soil sample containing *Schizothrix* sp., is an example of a compound produced by terrestrial cyanobacteria [20]. Recently, several anabaenopeptin variants were detected in the Nostocaelan genera *Nostoc*, *Desmonostoc* and *Brasilonema* isolated from plant leaves [21].

All of the above-mentioned anabaenopeptins (APs) are cyclic peptides comprised of a ring of five amino acid residues connected to an exocyclic residue through an ureido linkage. The general structure of anabaenopeptins is X_1_-CO-[Lys-X_3_-X_4_-MeX_5_-X_6_] [4], where the brackets indicate the cyclic part of the peptide, and X_1_ and X_3_ to X_6_ are variable amino acid residues. The structure contains both protein and non-protein amino acids. The peptide ring is derived from the cyclisation of the C-terminal carboxyl to the primary ε-amine of the *N*-terminal lysine. In the cyanobacterial anabaenopeptins, the ureido-bond and the d-Lys moiety are conserved in the structure, while the other residues vary and are in l-configuration. In the sponge-derived anabaenopeptins, both d- and l-configuration of the Lys residue occurs. A common feature of many APs is the presence of a homo-amino acid residue in position 4 and an *N-*methylated amino acid residue in position 5. An exception to this is demonstrated in the sponge-derived paltolides A–C where the residue in position 4 is l-Leu [22]. There are at least 87 published structures of anabaenopeptins isolated from cyanobacteria and nine sponge-derived anabaenopeptins. In the present paper, the anabaenopeptins with a letter code (such as anabaenopeptin A) are spellt out whereas those anabaenopeptins which are called by their molecular weight (such as AP 813) are abbreviated.

Anabaenopeptins are assembled by a non-ribosomal peptide synthetase (NRPS) enzyme complex, which has a modular structure. Each module contains specific functional domains responsible for adenylation and thiolation of the activated amino acid monomers, and elongation of the peptide sequence. The relaxed substrate specificity of NRPSs is considered to be the reason for the structural richness of peptides found in cyanobacteria [23]. The associated gene clusters can encode either a single starter module as in *Nostoc* and *Nodularia* or two starter modules as in *Anabaena* [24]. Several low molecular weight (MW) peptides synthetised via NRPS pathway are capable of inhibiting proteolytic enzymes, but the strength and specificity of the enzyme inhibition varies between the classes of peptides and the individual peptide variants. Characteristic for many anabaenopeptins is the inhibition of zinc-containing metalloexopeptidases such as carboxypeptidase A (CPA) and B (CPB) [14,25]. In experiments driven by pharmacological interests, anabaenopeptins have been shown to inhibit also carboxypeptidase U [26] and carboxypeptidase TAFIa, the thrombin activatable fibrinolysis inhibitor, important in the coagulation-fibrinolysis system [27]. Only a few studies so far have demonstrated activity of anabaenopeptins towards serine endopeptidases such as elastase, trypsin and chymotrypsin [28,29,30]. Anabaenopeptins have also been shown to inhibit serine/threonine protein phosphatases [31,32]. The biological role of anabaenopeptins is still uncertain, but theories include acting as a defense mechanism against parasites like chytrid fungi [33] and pathogenic amoeba [34], or to control cyanobacterial cell density [35].

In the present work, 14 anabaenopeptins were isolated from a bloom of Baltic Sea cyanobacteria consisting of *Nodularia*, *Aphanizomenon* and *Dolichospermum* spp. by preparative high performance liquid chromatography (HPLC). The putative structures of the peptides were determined by liquid chromatography coupled to tandem mass spectrometry (LC-MS/MS), and their activities towards carboxypeptidase A, protein phosphatase 1 and three proteolytic enzymes were tested.

## 2. Results and Discussion

In the brackish Baltic Sea, summer blooms of the filamentous and nitrogen-fixing cyanobacteria are frequently encountered. The blooms are mainly composed of toxic, nodularin-producing *Nodularia spumigena* and *Aphanizomenon flos-aquae,* which has been considered non-toxic. In coastal waters, but occasionally also in the open sea area, *Dolichospermum (Anabaena)* spp.—probable producers of microcystins—can be observed [36]. Our present study showed that bloom-forming Baltic Sea cyanobacteria, consisting mainly of *N. spumigena*, are an abundant source of bioactive cyanobacterial peptides.

The rough order of increasing hydrophobicity of the different peptide classes detected in the preliminary solid-phase extraction (SPE) fractionations of our sample was spumigins [4,5] (least hydrophobic, eluted with 20%–60% acidic methanol), aeruginosins [4,5] (eluted with 40%–60% acidic methanol), nodularins/microcystin-LR (eluted with 60%–80% acidic methanol) and anabaenopeptins (eluted with 60%–80% acidic methanol). Some of the anabaenopeptin variants have hydrophobicity equal to that of nodularin-R/microcystin-LR resulting in co-elution. The general oligopeptide pattern in the currently studied Baltic Sea cyanobacterial sample resembled that of *Nodularia* [8,37], which was expected since 50% of the material was *Nodularia*. Microcystin-LR originated most likely from *Dolichospermum* [36]. Baltic Sea *Aphanizomenon* has not been shown to produce anabaenopeptins (unpublished results by Mazur-Marzec, and [38]) although anabaenopeptins I and J were originally isolated from Japanese *Aphanizomenon flos-aquae* [14]. Anabaenopeptins were present both in the 60% and 80% SPE fractions. The previously discovered anabaenopeptin E (*m*/*z* 851), AP 841 (*m*/*z* 842) [37], and a new anabaenopeptin with *m*/*z* 824, with the planar structure Ile + CO[Lys + Val + Hph + MeHty + Ser] were detected in the 60% SPE fraction but only in low amounts. During the first preparative HPLC purification (System 1) of the 80% SPE fraction containing most of the APs, 15 time segments were collected, each of which was further purified in the second preparative HPLC system. The second preparative HPLC (System 2) resulted in 18 fractions containing one or several APs each. Seventeen anabaenopeptins were detected eight of which were regarded as new ones. Five of them were isolated in a purified form without coelution of other anabaenopeptins or nodularin. The isolated peptides with their observed *m*/*z* values, putative planar structures and approximative amounts are listed in Table 1. The concentrations of the anabaenopeptins were calculated by comparing the peak areas at 214 nm with the mean peak areas of the standards anabaenopeptin A and B (both 10 μg/mL).

**Figure 1:**
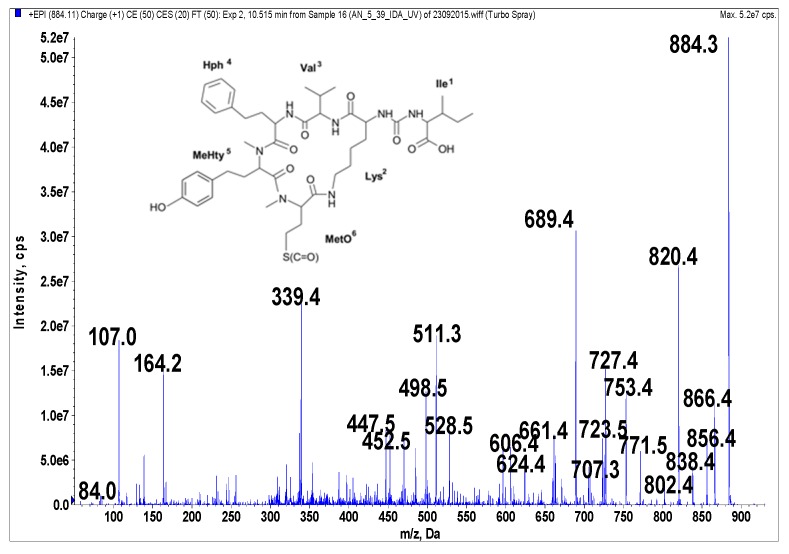
Schematic structure and mass fragmentation spectrum of anabaenopeptin NP 883. The structure of the peptide (Ile + CO[Lys + Val + Hph + MeHty + MetO]) was deduced on the basis of the following fragments: 866 [M + H − H_2_O], 856 [M + H − CO], 838 [M + H − CO − H_2_O], 820 [M + H − CH_3_SOH (from MetO)], 802 [M + H − CH_3_SOH − H_2_O), 771 [M + H − Ile], 753 [M + H − Ile − H_2_O], 727 [M + H − (Ile + CO)], 723 [M + H − Hph], 707 [M + H − Ile − CH_3_SOH], 689 [M + H − Ile − CH_3_SOH − H_2_O], 624 [M + H − (Hph + Val)], 606 [M + H − (Hph + Val) − H_2_O], 528 [M + H − Ile − Hph − CH_3_SOH − H_2_O], 511 [M + H − Ile − (Hph + Val)], 452 [MeHty + Hph + Val + H], 339 [MetO + MeHty + H], 447 [M + H − Ile − (Hph + Val) − CH_3_SOH], 164 MeHty, 107 [CH_2_PhOH], 84 Lys-immonium ion.

**Figure 2:**
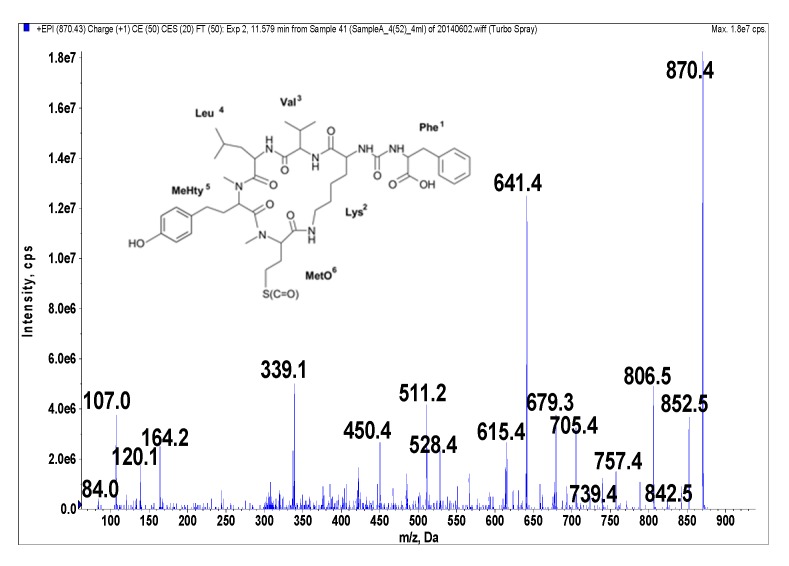
Schematic structure and mass fragmentation spectrum of anabaenopeptin NP 869. The structure of the peptide (Phe + CO[Lys + Val + Leu + MeHty + MetO]) was deduced on the basis of the following fragments: 852 [M + H − H_2_O], 842 [M + H − CO], 806 [M + H − CH_3_SOH (from MetO)], 788 [M + H − CH_3_SOH − H_2_O), 757 [M + H − Leu], 739 [M + H − Leu − H_2_O], 705 [M + H − Phe − H_2_O], 679 [M − MeHty + H], 641 [M + H − Phe − CH_3_SOH − H_2_O], 615 [M − MeHty + H − CH_3_SOH], 528 [M + H − Phe − Leu − CH_3_SOH − H_2_O], 511 [M + H − Phe − (Leu + Val)], 339 [MetO + MeHty + H], 164 MeHty, 120 Phe-immonium ion, 107 [CH_2_PhOH], 84 Lys-immonium ion.

**Figure 3:**
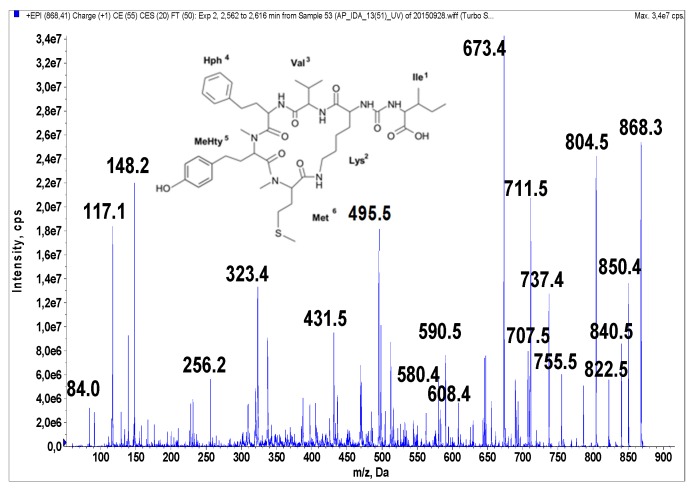
Schematic structure and mass fragmentation spectrum of anabaenopeptin NP 867. The structure of the peptide (Ile + CO[Lys + Val + Hph + MeHty + Met]) was deduced on the basis of the following fragments: 850 [M + H − H_2_O], 840 [M + H − CO], 822 [M + H − CO − H_2_O], 755 [M + H − Ile], 737 [M + H − Ile − H_2_O], 711 [M + H − (Ile + CO)], 707 [M + H − Hph], 693 [M + H − (Ile + CO) − H_2_O], 608 [M + H − (Hph + Val)], 590 [M + H − (Hph + Val) − H_2_O], 580 [M + H − (Hph + Val) − CO], 495 [M + H − Ile − (Hph + Val)], 323 [MeHty + Met + H], 84 Lys-immonium ion.

**Figure 4:**
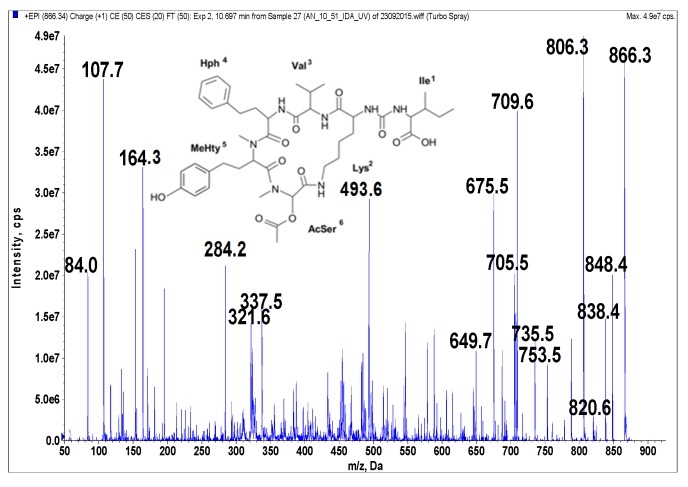
Schematic structure and mass fragmentation spectrum of anabaenopeptin NP 865. The structure of the peptide (Ile + CO[Lys + Val + Hph + MeHty + AcSer]) was deduced on the basis of the following fragments: 848 [M + H − H_2_O], 838 [M + H − CO], 820 [M + H − CO − H_2_O], 806 [M + H − CH_3_COOH (from AcSer)], 753 [M + H − Ile], 735 [M + H − Ile − H_2_O], 709 [M + H − (CO + Ile)], 705 [M + H − Hph], 675 [M + H − Ile − CH_3_COOH − H_2_O], 649 [M + H − (CO + Ile) − CH_3_COOH], 493 [M + H − Ile − (Hph + Val)], 337 [M + H − Ile − (Hph + MeHty) − CH_3_COOH], 321 [MeHty + AcSer + H], 164 MeHty, 107 [CH_2_PhOH], 84 Lys-immonium ion.

**Figure 5:**
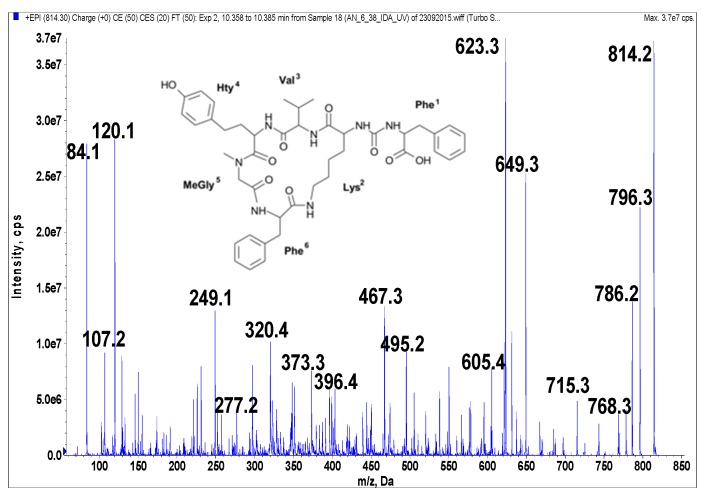
Schematic structure and mass fragmentation spectrum of anabaenopeptin AP 813. The structure of the peptide (Phe + CO[Lys + Val + Hty + MeGly + Phe]) was deduced on the basis of the following fragments: 796 [M + H − H_2_O], 786 [M + H − CO], 768 [M + H − CO − H_2_O], 715 [M + H − Val], 649 [M + H − Phe − H_2_O], 623 [M + H − (CO + Phe)], 605 [M + H − (CO + Phe) − H_2_O], 495 [Phe + MeGly + Hty + Val + H], 467 [Phe + Lys + CO + Phe + H], 396 [Hty + MeGly + Phe + H], 373 [M + H − Phe − (Hty + Val) − H_2_O], 320 [M + H − Phe − (Val + Hty + MeGly + Phe)], 277 [Hty + Val + H], 249 [Hty + MeGly + H], 120 Phe-immonium ion, 107 [CH_2_PhOH], 84 Lys-immonium ion.

Anabaenopeptins were identified and their putative structures deduced from the LC-MS/MS fragmentation spectra by comparison to already known peptides and their fragment ions [39,40,41]. LC-MS/MS cannot distinguish between isomeric amino acids or provide information on stereochemistry. The stereochemistry analyses will need further study, and the isolated compounds may have different isomers. The real compounds responsible for the activity will be confirmed after stereochemistry elucidation by NMR or amino acid analysis. In addition, the presence of Leu cannot be distinguished from that of Ile. When it comes to Leu and Ile, the putative structural assignments of the peptides are based on analogy with earlier known structures. Under the conditions used in this study, fragmentation spectra with a high number of product ions were obtained (Figure 1, Figure 2, Figure 3, Figure 4 and Figure 5). The identification of the peptide structures was based on the existing information about the known anabaenopeptin components and their location in the molecule. The ion signals occurring in the spectra at low *m*/*z* values indicated the presence of specific units, e.g., Lys (*m*/*z* 84, 129), MeHty (*m*/*z* 107, 164) or Phe (*m*/*z* 120). The presence and sequence of the units was confirmed by the loss of respective residues from molecular ion and from fragment ions (e.g., *m*/*z* 128 for Lys, *m*/*z* 191 for MeHty, *m*/*z* 147 for Phe). In anabaenopeptins with AcSer or MetO in position 6, a loss of 60 Da or 64 Da, respectively, from pseudomolecular ion and some other fragment ions was usually observed. These units correspond to the loss of CH_3_COOH from AcSer and CH_3_SOH from MetO. For example, in the fragmentation spectrum of NP 865 (Figure 4), which contains AcSer, an ion signal at *m*/*z* 806 is observed, while the spectrum of NP 883, with MetO in position 4, is characterized by the presence of a high intensity ion signal at *m*/*z* 820 (Figure 1). In the process of structure elucidation, a series of *b* and *y*-ions formed by the cleavage of peptide bonds, as well as *a*-ions formed by the loss of carbonyl group (CO), provided the most valuable information on the sequence of residues in the peptides.

Most of the new anabaenopeptins in the sample belonged to the subclass of nodulapeptins (NPs) typical for *N. spumigena* harboring Met or MetO (*m*/*z* 900, 856, 884 and 868) and Ser or AcSer (*m/z* 858 and 866) in position 6. Nodulapeptin of *m*/*z* 884 (NP 883) was the main compound with a final yield of 0.022% (10.9 mg peptide per 50 g freeze-dried cyanobacterial material). Other major anabaenopeptins in the sample included anabaenopeptin D, anabaenopeptin A, oscillamide Y (Osc Y) as well as NP 933 (*m*/*z* 934) detected recently [37]. Three compounds with the same molecular weight of 899 (*m*/*z* 900) were detected, two of them previously known [24,42]. The new compound with *m*/*z* 900 in the bloom sample contained Hty in position 4 and MetO in position 6. This compound differed from the partly co-eluting *m*/*z* 934 by one residue, having Ile in position 1 instead of Phe.

Nine of the 17 anabaenopeptins in the sample contained Phe and six had Ile in the exocyclic position. Since the strains of *Nodularia* have been suggested to produce nodulapeptins with only one kind of residue in the exocyclic position, Ile or Phe [23,37], we assume that at least two different strains of *Nodularia* were present in the bloom, one producing nodulapeptins with Ile and another producing nodulapeptins with Phe in the exocyclic position. Anabaenopeptins A, D and Osc Y are most often isolated from *Dolichospermum* and *Planktothrix.* Mazur-Marzec *et al.* [37] were the first to report of anabaenopeptin D and AP 841, anabaenopeptins, other than nodulapeptins in *Nodularia* strains isolated from the Baltic Sea. The compound AP 841 (*m*/*z* 842) was also detected in the present sample. The origin of the new compound AP 813 (*m*/*z* 814) remained unclear. AP 813 has Phe in the exocyclic position and contains *N-*MeGly in position 5. Anabaenopeptins containing *N-*MeGly have been previously isolated from *Dolichospermum* [43] and *Nostoc* [24].

**Table 1:** Isolated anabaenopeptins.

Fraction *	Amount of Isolated Anabaenopeptin (μg)	*m*/*z* and Proposed Structure of Anabaenopeptin Components in Fractions	Anabaenopeptin Concentration in Tested Samples (µg/mL)	PP1 IC_50_ (ng/mL)	Carboxypeptidase A IC_50_ (µg/mL)	NOD Concentration in Tested Samples (ng/mL)	References
1(38)	1305	934 Phe + CO[Lys + Val + Hty + MeHty + MetO]	43.5	16	20	-	[37]
2(46)	1287	844 (Anabaenopeptin A)	42.9	86	<3	43.4	[6]
		Tyr + CO[Lys + Val + Hty + MeAla + Phe]					
2(48)	273	844 (Anabaenopeptin A)	9.1	88	<3	12.9	[6]
		Tyr + CO[Lys + Val + Hty + MeAla + Phe]					
2(50)	129	900 Ile + CO[Lys + Val + Hty + MeHty + MetO]	4.3	40	<3	4.8	New, this study
		934 Phe + CO[Lys + Val + Hty + MeHty + MetO]					[37]
		856 Phe + CO[Lys + Val + Val + MeHty + MetO]					New, this study
4(50)	1305	858 (Oscillamide Y)	43.5	62	15	9.0	[7]
		Tyr + CO[Lys + Ile + Hty + MeAla + Phe]					
4(51)	1305	858 (Oscillamide Y)	43.5	62	15	-	[7]
		Tyr + CO[Lys + Ile + Hty + MeAla + Phe]					
4(52)	51	870 Phe + CO[Lys + Val + Leu + MeHty + MetO]	1.7	66	-	-	New, this study
5(39/40)	10,914	884 Ile + CO[Lys + Val + Hph + MeHty + MetO]	181.9	53	<3	-	New, this study
6(38)	123	814 Phe + CO[Lys + Val + Hty + MeGly + Phe]	4.1	435	<4	-	New, this study
6(39)	252	916 Phe + CO[Lys + Val + Hty + MeHty + AcSer]	8.4	50	<4	2.6	[37]
7(39)	87	808 Ile + CO[Lys + Ile + Hty + MeAla + Phe]	2.9	55	<25	1.5	[37]
8(40)	2733	828 (Anabaenopeptin D)	91.1	53	<3	-	[10]
		Phe + CO[Lys + Val + Hty + MeAla + Phe]					
8(44/45)	3816	918 Phe + CO[Lys + Val + Hph + MeHty + MetO]	63.6	100	<3	-	[42]
9(44)	69	900 Ile + CO[Lys + Met + Hph + MeHty + Met]	2.3	60	<28	-	[24]
9(45)	108	858 Phe + CO[Lys + Val + Hph + MeHty + Ser]	3.6	50	<20	-	New, this study
		918 Phe + CO[Lys + Val + Hph + MeHty + MetO]					[42]
10(51)	579	866 Ile + CO[Lys + Val + Hph + MeHty + AcSer]	19.3	435	35	-	New, this study
10(52)	84	900 Phe + CO[Lys + Val + Hph + MeHty + AcSer]	2.8	140	<22	-	[42]
13(51)	741	868 Ile + CO[Lys + Val + Hph + MeHty + Met]	24.7	71	45	-	New, this study

* Sample number(retention time).

### Activity Studies of the Anabaenopeptins

All isolated fractions containing anabaenopeptins inhibited carboxypeptidase A (CPA; with the exception of one peptide variant which was present at a low concentration) and protein phosphatase 1 (PP1). The activities of the isolated anabaenopeptins are shown as IC_50_ values in Table 1 (determined by probit analysis as well as a graphical method using dose-response curves). Table 1 is useful for the comparison of the inhibitory activities of the peptides isolated in this study but cannot be directly used for a comparison with previous studies, as the IC_50_ values of the enzyme inhibitors vary depending on the assay parameters such as the nature of the enzyme, the used substrate and other experimental conditions.

The peptides showed activity with IC_50_ values from 16 to 435 ng/mL in the PP1 assay and from below 3 to 45 μg/mL in the CPA assay. Anabaenopeptin 869 in fraction 4(52) did not inhibit the carboxypeptidase A at the tested concentration. The activities of the fractions consisting of more than one anabaenopeptin are the summed effect of the anabaenopeptins present. Acquired standards of anabaenopeptins A, B and J as well as Osc Y were tested for activity towards carboxypeptidase A and protein phosphatase 1. All four compounds showed inhibition of PP1. Anabaenopeptin J and Osc Y were additionally active against CPA. The presence of nodularin complicated the interpretation of enzymatic activities towards PP1 in some fractions. No anabaenopeptins isolated in this study induced inhibition of elastase, trypsin or thrombin, irrespective of the residue in the exocyclic position (Phe, Ile and Tyr).

Substitutions of the amino acid residues are thought to determine the degree of protease inhibition as well as the protease specificity of the compounds [44]. Recent computational docking studies have shown the importance of the aliphatic part of the d-Lys and its stereochemistry as well as the importance of the residues adjacent to the urea bond in interacting with the active binding pocket of carboxypeptidase A [27,45]. Thirteen of the anabaenopeptins presented in Table 1 have the aromatic residue Phe, five have Ile and four Tyr in the exocyclic position. The CPA inhibition activities of all these anabaenopeptins irrespective of the amino acid in position 1 varied from weak to more potent. Thus, more than one element of the structure has an effect on the activity of anabaenopeptins. Anabaenopeptins with exocyclic Ile and Tyr have been shown to inhibit CPA strongly with IC_50_ values of 0.0052–0.022 μg/mL (hippur-l-phenylalanine as a substrate) [14,25,46]. The CPA inhibition by anabaenopeptin H having exocyclic Arg was shown to be a weaker although still a potent inhibitor with IC_50_ of 3.4 μg/mL [14]. No Arg-containing anabaenopeptins were among the isolated and tested anabaenopeptins in our sample, although some anabaenopeptin E was detected during the purification. We have been unable to locate literature on anabaenopeptins with Phe in the exocyclic position inhibiting CPA so far. Our work demonstrates for the first time the ability of nodulapeptins with either Ile or Phe in the exocyclic position to inhibit CPA.

The cyclic hepta- and pentapeptides, microcystins and nodularins, are the most extensively studied toxic secondary metabolites of cyanobacteria. Microcystins and nodularins exhibit their toxicity by inhibition of protein serine/threonine phosphatases type 1 (PP1) and 2A (PP2A) [47]. Microcystins and nodularins are considered to be potent PP1 inhibitors, with reported IC_50_-values of 1.1–1.9 nM [48,49]. All isolated anabaenopeptins presented in Table 1 inhibited protein phosphatase 1 but clearly with lower potencies than the toxins. The influence of nodularin to the PP1 inhibition cannot be excluded when present in the fraction.

The comparison of the PP1 inhibition of different anabaenopeptins in our sample with previously discovered anabaenopeptins is hampered by the low number of studies performed on this issue. The inhibition of PP1 (from rabbit skeletal muscle) by anabaenopeptins has been reported for oscillamides B, C, and anabaenopeptin F [31] as well as for anabaenopeptin A and B [32]. The presence of Arg in the exocyclic position in all these active compounds were deduced to be linked to the inhibitory activity. Osc Y with *N*-MeAla at position 5 and exocyclic Tyr was considered only a weak inhibitor of PP1 (no effect even at 100 μg/mL) compared to oscillamide B, C and anabaenopeptin F. The IC_50_ value was reported only for Osc C which was 0.9 μM [31]. In our study, Osc Y showed inhibitory activity with IC_50_ of 62 ng/mL which was higher than that of anabaenopeptin A, with two isomeric forms chromatographed in fractions 2(46) and 2(48) and coeluting with nodularin. The IC_50_ values of the isomeric forms of anabaenopeptin A were 86 and 88 ng/mL. No Arg-containing anabaenopeptins were isolated from our sample. Our results showed that all of the 14 isolated APs and NPs inhibited protein phosphatase 1.

## 3. Experimental Section

### 3.1. Reagents

Water was purified to 18.2 MΩ cm on a Milli-Q Synthesis system (Molsheim, France). Methanol, HPLC gradient grade, was from Merck (Darmstadt, Germany). Acetic acid, formic acid, trifluoroacetic acid and LC-MS grade acetonitrile were purchased from Sigma-Aldrich (St. Louis, MO, USA).

The following reagents used for enzymatic assays were purchased from Sigma-Aldrich: trypsin (product number T0303), thrombin bovine plasma (T4648), elastase (E0258), carboxypeptidase A (C9268), aprotinine (A6103), *N*-p-tosyl-Gly-Pro-Lys-p-nitroanilide acetate salt (T6140), *N*-Succinyl-Gly-Gly-Phe-p-nitroanilide (S1899), 4-(2-Aminoethyl) benzenesulfonyl fluoride hydrochloride—AEBSF (A8456), elastatinal (E0881), *N*-Succinyl-Ala-Ala-Ala-p-nitroanilide (S4760), carboxypeptidase inhibitor—CPI (C0297), p-nitrophenyl phosphate disodium salt hexahydrate—p-NPP (71768), albumin bovine—BSA (A4503) and dl-dithiothreitol DTT (D0632).

Protein phosphatase 1—PP1 was from New England Biolabs (Ipswich, MA, USA). *N*_α_-benzoyl-l-arginine 4-nitroanilide hydrochloride—BAPNA (ACROS 227740050) was from Acros Organics (Geel, Belgium). *N*-(4-methoxyphenylazoformyl)-Phe-OH (M-2245.0100) was purchased from Bachem (Bubendorf, Switzerland). In addition, the following analytical reference materials were used: CRM-Nodularin (IBM-NRC, Halifax, NS, Canada), anabaenopeptin A and B (Enzo Life Sciences, Lausen, Switzerland), anabaenopeptin J and oscillamide Y (Cyano Biotech, Berlin, Germany).

### 3.2. Sampling and Extraction of the Cell Material

Cyanobacterial material was collected with a plankton net (100 µm mesh size) from the Gulf of Gdansk, close to Gdynia-Redłowo, on 5 July 2012. The cyanobacterial community in the bloom sample was composed of *Nodularia spumigena* (50% of the cyanobacterial biomass), *Aphanizomenon flos-aquae* (40%) and *Dolichospermum* spp. (10%). The freeze-dried material (50 g) was extracted with 5% acetic acid in water (500 mL) by 5-min probe sonication (HD2070 Sonopuls ultrasonic disrupter, Bandelin, Berlin, Germany) followed by 15 min bath sonication. The extract was centrifuged (10,000 rpm, 15 min) and the remaining residue was re-extracted three times with 500 mL of 5% (*v/v*) acetic acid. Additionally, two extractions were done with 500 mL of 60% methanol in water. The methanolic extracts were combined, evaporated in vacuum evaporator with water bath (35 °C) and dissolved in 300 mL of 5% acetic acid. Finally, all the extracts were combined, centrifuged (10,000 rpm, 15 min) and filtered through Whatman glassfibre filters, type GF/C (Maidstone, UK) discs. The total volume of the extract was 2.3 L.

### 3.3. Fractionation of Peptides by Solid-Phase Extraction (SPE)

For the first SPE, the extract was divided in aliquots of approx. 550 mL, and applied on six Sep–Pak Vac 10 g 35cc cartridges with tC_18_ resin (Waters, Milford, MA, USA). The adsorbed substances were eluted from each cartridge with 120 mL of 75% methanol in water and the eluates were rotary evaporated to dryness. The residues were dissolved in a total volume of 300 mL of 5% acetic acid for the second extraction step on six 6-g OASIS HLB (Hydrophilic-Lipophilic-Balanced) cartridges (Waters). The material adsorbed to each cartridge was sequentially eluted and fractionated with 20% (100 mL), 40% (300 mL), 50% (250 mL) and 80% (500 mL) methanol in water with 0.1% (*v/v*) formic acid (FA). The fractions eluted with 80% methanol with 0.1% FA, which contained the majority of the peptides as monitored by LC-MS/MS, were combined and evaporated to dryness and dissolved in 150 mL of 5% acetic acid. This solution was subjected to further purification (third SPE) using three 1-g HLB cartridges. Sequential elution was performed with 50 mL of 20%, 30%, 40%, 50% and 60% methanol containing 0.1% (*v/v*) FA and then with 300 mL of 80% methanol containing 0.1% FA. The 80% methanolic eluates with 0.1% FA, containing anabaenopeptins, were combined and evaporated in a rotary evaporator. The dry residue was dissolved in 10 mL of 15% methanol, centrifuged and the supernatant was filtered through a Pall (Ann Arbor, MI, USA) GF/C-GHP (polypropylene membrane with a built-in glassfibre prefilter) Acrodisc 25 mm syringe filter. Dilutions of the resulting solution and the purchased standard peptides were used to optimize the chromatographic conditions in analytical and preparative HPLC as well as the mass spectrometric conditions.

### 3.4. Monitoring of Peptides by Analytical HPLC

The analytical separations were carried out on a Agilent (Waldbronn, Germany) 1100 Series HPLC system consisting of degasser, quaternary pump, thermostated column compartment at 40 °C and diode-array detector. The peptides were monitored at 214 nm, 238 nm and 280 nm and UV spectra were recorded at 200–300 nm. Separation was achieved on a Synergi 2.5 μm Polar-RP 100 A, 100 mm × 3.0 mm I.D. (Phenomenex, Torrance, CA, USA) column with a compatible guard column. The mobile phase system consisted of A: 0.05% trifluoroacetic acid (TFA) in water and B: 0.05% TFA in acetonitrile. The following linear gradient was employed: 0 min 10% B, 20 min 60% B, 21 min 60% B and 21.1 min 10% B. Flow-rate was 0.5 mL·min^−1^. The concentrations of the anabaenopeptins were calculated by comparing the peak-areas at 214 nm with the mean peak areas of the standards anabaenopeptins A and B (both 10 μg·mL^−1^).

### 3.5. Isolation of Anabaenopeptins with Dual Preparative HPLC

The preparative HPLC system consisted of Merck-Hitachi (Tokyo, Japan) LaChrom Series HPLC pump with a 10 mL injection loop and the photodiode-array detector set at 270 nm. A CTO-10AV column oven (Shimadzu, Suzhou, China) was thermostated at 40 °C. The first step in the purification of anabaenopeptins (System 1) was conducted on a Synergi 4 μm Polar-RP 80 A, 250 mm × 10 mm, column (Phenomenex, Torrance, CA, USA). The mobile phase system consisted of A: 0.05% TFA in water and B: 0.05% TFA in acetonitrile. The gradient was from 32% B to 42% B over 80 min. The flow-rate was initially 3 mL·min^−1^ but it was increased to 4 mL·min^−1^ at 3 min. 0.5 min fractions (2 mL) were collected by a fraction collector (Gilson, Middleton, MI, USA).

The second preparative HPLC system (System 2) was as depicted above except for the solvent system which consisted of A: ammonium acetate in water (15 g/L)—acetonitrile (ACN) (95:5) and B: ammonium acetate in water (30 g/L)—ACN (50:50). The gradient was from 20% B to 95% B over 80 min, increasing to 100% B at 85 min and back to 20% B at 98 min. The flow-rate was as given above.

The 10 mL of sample containing anabaenopeptins dissolved in 15% methanol was separated according to the preparative System 1. 0.5-min fractions were collected. All fractions were analyzed by flow injection analysis—MS/MS (FIA-MS/MS). Fractions containing anabaenopeptins were diluted with water and concentrated on 400 mg OASIS HLB cartridges. The peptides were eluted with 2 + 2 mL of 100% methanol. Aqueous dilutions were made of the SPE eluates and analysed both by analytical HPLC (Synergi Polar column TFA-ACN mobile phase) and FIA-MS/MS. The eluates containing the compounds of interest (15 samples) were evaporated to nearly dryness and each sample was dissolved in 3 mL of 15% methanol containing ammonium acetate (1.5 g/100 mL) before separation in the preparative System 2. This time 1 min fractions were collected. Fractions containing anabaenopeptins were diluted and analysed by HPLC and FIA-MS/MS. The purified fractions were desalted and concentrated on 200 mg or 400 mg OASIS HLB cartridges for further testing in various enzymatic assays.

### 3.6. LC-MS/MS

The structures of the cyanobacterial peptides were characterized by Applied Biosystems Sciex (Concorde, ON, Canada) QTRAP 5500 LC-MS/MS as described by Mazur-Marzec *et al.* (2013) [37], but the collision energy (CE) was set at 50 V. In the samples subjected to enzymatic assays, the presence of nodularin was additionally analyzed using multiple reaction monitoring mode with the following transition ions 825 → 135 (quantifier, CE 60), 825 → 389 (CE 55), 825 → 227 (CE 55). The detection limit of nodularin in MRM mode was 0.2 ng/mL (5 μL sample injected).

### 3.7. FIA-MS/MS

The FIA-MS/MS experiments were carried out on an Agilent Technologies (Waldbronn, Germany) 1200 Rapid Resolution (RR) LC coupled to a Bruker Daltonics HCT Ultra Ion trap MS (Bremen, Germany). The ion trap MS was operated in the positive electrospray ion mode. The Ion Charge Control (ICC) target was set to 200,000 with a maximum accumulation time of 200 ms. The capillary voltage was set at 4.0 kV. The ion source parameters were set as follows: dry temperature 350 °C, nebulizer pressure 30 psi and dry gas flow 8.0 L/min. The mobile phase consisted of solvents A: 99% water—1% ACN—0.1% FA and B: ACN—0.1% FA. Flow injection analysis was performed under isocratic conditions with 50% B.

### 3.8. Enzymatic Assays

APs were assayed for bioactivity by protein phosphatase 1, carboxypeptidase A, trypsin, elastase and thrombin inhibition assays as described in Mazur-Marzec *et al.* 2015 [50]. Some details of the performed assays are presented in Table 2.

**Table 2:** Conditions under which the protein phosphatases and proteases inhibition assays were performed (*p*-NPP 4-nitrophenyl phosphate disodium salt hexahydrate; BAPNA *N*_α_-benzoyl-l-arginine 4-nitroanilide hydrochloride; AEBSF 4-(2-aminoethyl)benzenesulfonyl fluoride hydrochloride).

Inhibition Assay	Substrate	Inhibitor	Preincubation Time (min)	Reaction Time (min)	Reaction Temperature (°C)	Wave Length (nm)
Protein phosphatase (PP1)	*p*-NPP	Nodularin	-	120	37	405
		
Trypsin	BAPNA	Aprotinine	15	5	25	405
Thrombin	*N*-*p*-tosyl-Gly-Pro-Lys-*p*-nitroanilide acetate salt	AEBSF	10	-	37	405
Elastase	2 mM of *N*-succinyl-Ala-Ala-Ala-p-nitroanilide	Elastatinal	15	10	25	405
Carboxypeptidase A	*N*-(4-methoxy-phenyl-azoformyl)-Phe-OH	Carboxypeptidase inhibitor from potato tuber (CPI)	5	15	37	350

## 4. Conclusions

A bloom sample of Baltic Sea cyanobacteria comprised of *Nodularia*, *Dolichospermum* and *Aphanizomenon* was extracted and fractionated. Fourteen anabaenopeptins were isolated and determined structurally by LC-MS/MS. Most of the isolated compounds were nodulapeptins, a subclass of anabaenopeptins typical for *N. spumigena.* Eight novel anabaenopeptins were discovered, five isolated with no co-eluting peptides. All anabaenopeptins showed activity towards PP1 and carboxypeptidase A (with the exception of one variant), but no inhibition against chymotrypsin, trypsin and thrombin was observed.

## Supplementary Files

Supplementary File 1
