# Supplementary material for: Structures and Activity of New Anabaenopeptins Produced by Baltic Sea Cyanobacteria
Source: Mar Drugs. 2015 Dec 30;14(1):8. doi: 10.3390/md14010008 (PMC4728505; doi:10.3390/md14010008)
Supplement: Supplementary File 1 [file marinedrugs-14-00008-s001.pdf]

# Supplementary Material

# Structure and Activity of Anabaenopeptins Produced by Baltic Sea Cyanobacteria

**Lisa Spoof [1], Agata Błaszczyk [2], Jussi Meriluoto [1], Marta Cegłowska [2] and Hanna Mazur-Marzec [2]**

[1] Biochemistry, Faculty of Science and Engineering, Åbo Akademi University, Tykistökatu 6A, 20520 Turku, Finland; lspoof@abo.fi (L.S.); jussi.meriluoto@abo.fi (J.M.)

[2] Department of Marine Biotechnology, University of Gdańsk, Al. Marszałka Piłsudskiego 46, 81-378 Gdynia, Poland; agata.blaszczyk@ug.edu.pl (A.B.); ma.ceglowska@gmail.com (M.C.); biohm@ug.edu.pl (H.M.-M.)

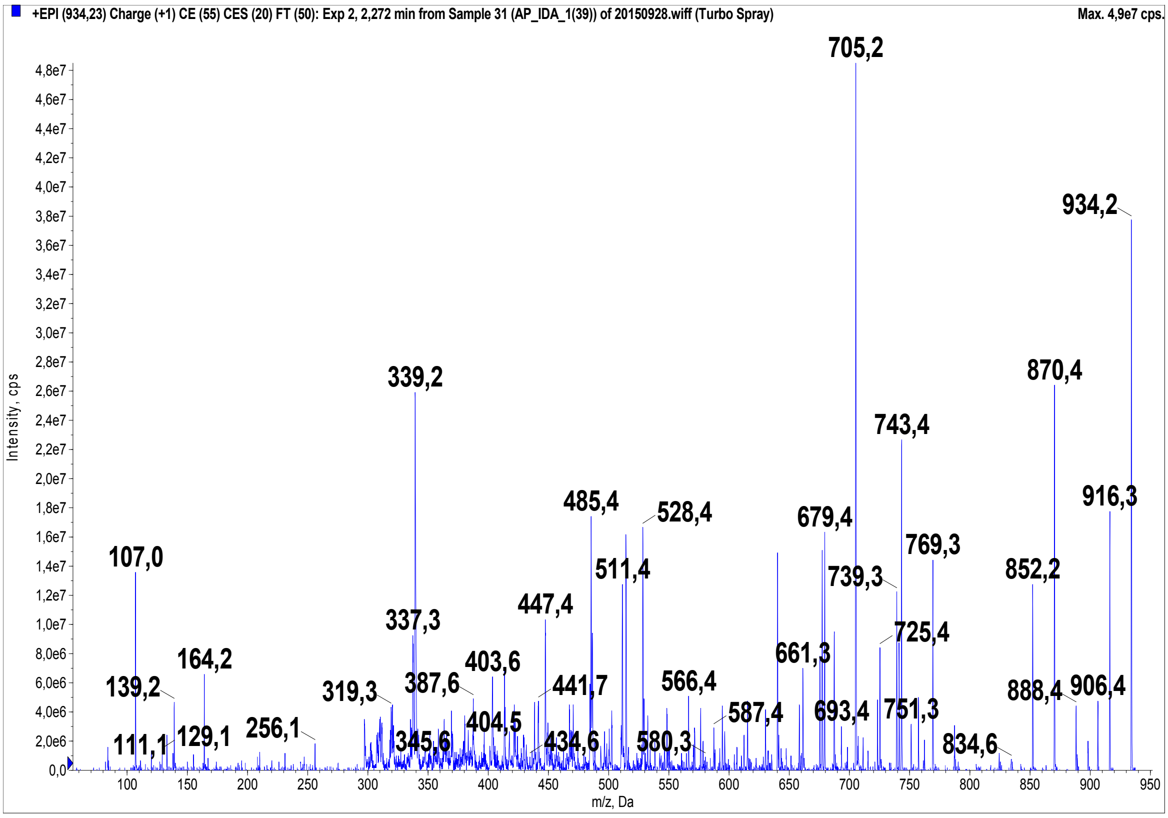


**Figure S1.** Mass fragmentation spectrum of anabaenopeptin with *m*/*z* of $[M + H]^+$ at 934 and structure Phe + CO + [Lys + Val + Hty + MeHty + MetO] elucidated on the basis of following fragments: 916 [M + H − $H_2O$], 906 [M + H − CO], 888 [M + H − $H_2O$ − CO], 870 [M + H − $CH_3SOH$ (from MetO)], 852 [M + H − $CH_3SOH$ − $H_2O$], 769 [M + H − Phe − $H_2O$], 743 [M + H − (CO + Phe)], 705 [M + H − Phe − $CH_3SOH$ − $H_2O$], 679 [M + H − (CO + Phe) − $CH_3SOH$], 661 [M + H − (CO + Phe) − $CH_3SOH$ − $H_2O$], 566 [M + H − (Hty + MHty)], 528 [M + H − Phe − Hty − $CH_3SOH$ − $H_2O$], 511 [M + H − Phe − (Hty + Val)], 447 [M + H − Phe − (Hty + Val) − $CH_3SOH$], 403 [MetO + Lys(CO) + Val + H], 339 [MetO + MeHty + H],164 MeHty, 107 [$CH_2PhOH$], 84 Lys–immonium ion.

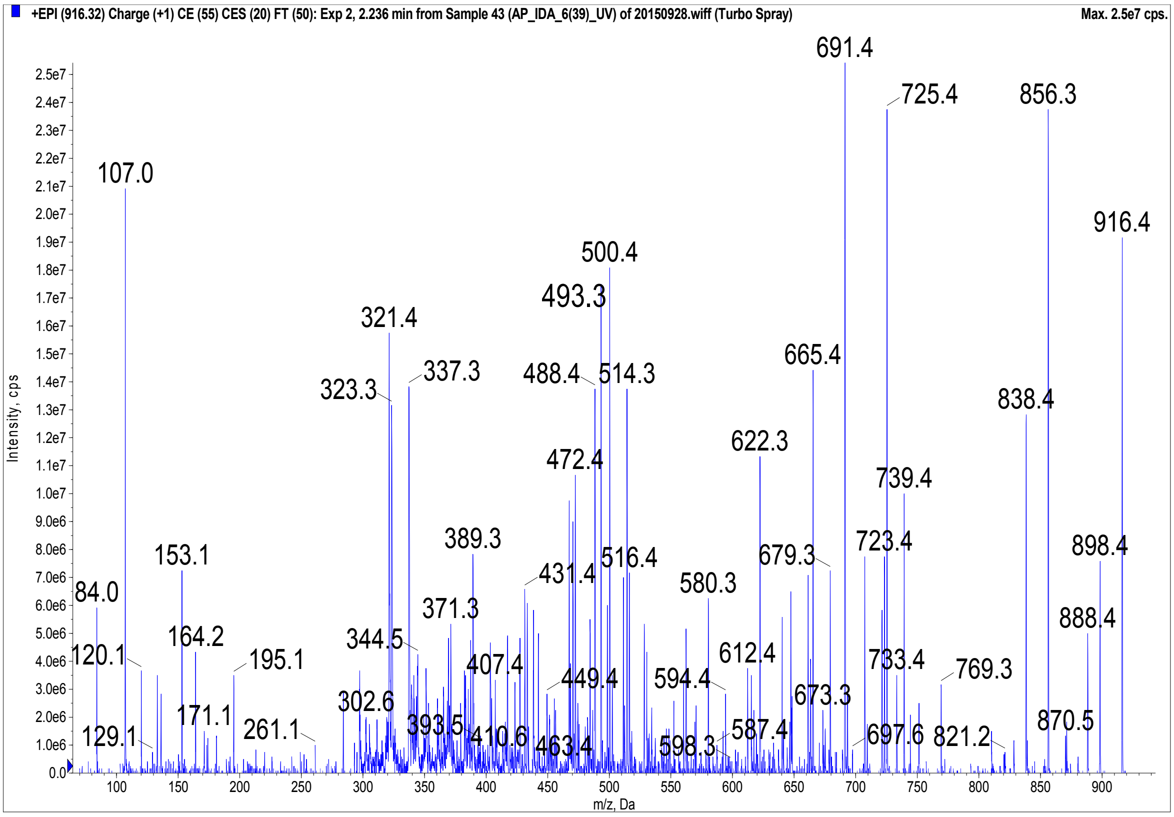


**Figure S2.** Mass fragmentation spectrum of anabaenopeptin with *m*/*z* of $[M + H]^+$ at 916 and structure Phe + CO + [Lys + Val + Hty + N − MeHty + AcSer] elucidated on the basis of following fragments: 898 [M + H − $H_2O$], 888 [M + H − CO], 870 [M + H − CO − $H_2O$], 856 [M + H − $CH_3COOH$ (from AcSer)], 769 [M + H − Phe], 739 [M + H − Hty], 725 [M + H − MHty] or [M + H − (CO + Phe)], 665 [M + H − (CO + Phe) − $CH_3COOH$], 640 [M + H − (Val + Hty)], 622 [M + H − (Val + Hty) − $H_2O$], 612 [M + H − (Val + Hty) − CO], 594 [M + H − (Val + Hty) − CO − $H_2O$], 493 [M + H − Phe − (Hty + Val)], 488 [Val + Lys + AcSer + MeHty − $CH_3COOH$ + H], 389 [Lys + (AcSer − $CH_3COOH$) + MeHty + H], 321 [MeHty + AcSer + H], 164 MeHty, 120 Phe immonium ion, 107 [$CH_2PhOH$], 84 Lys–immonium ion.

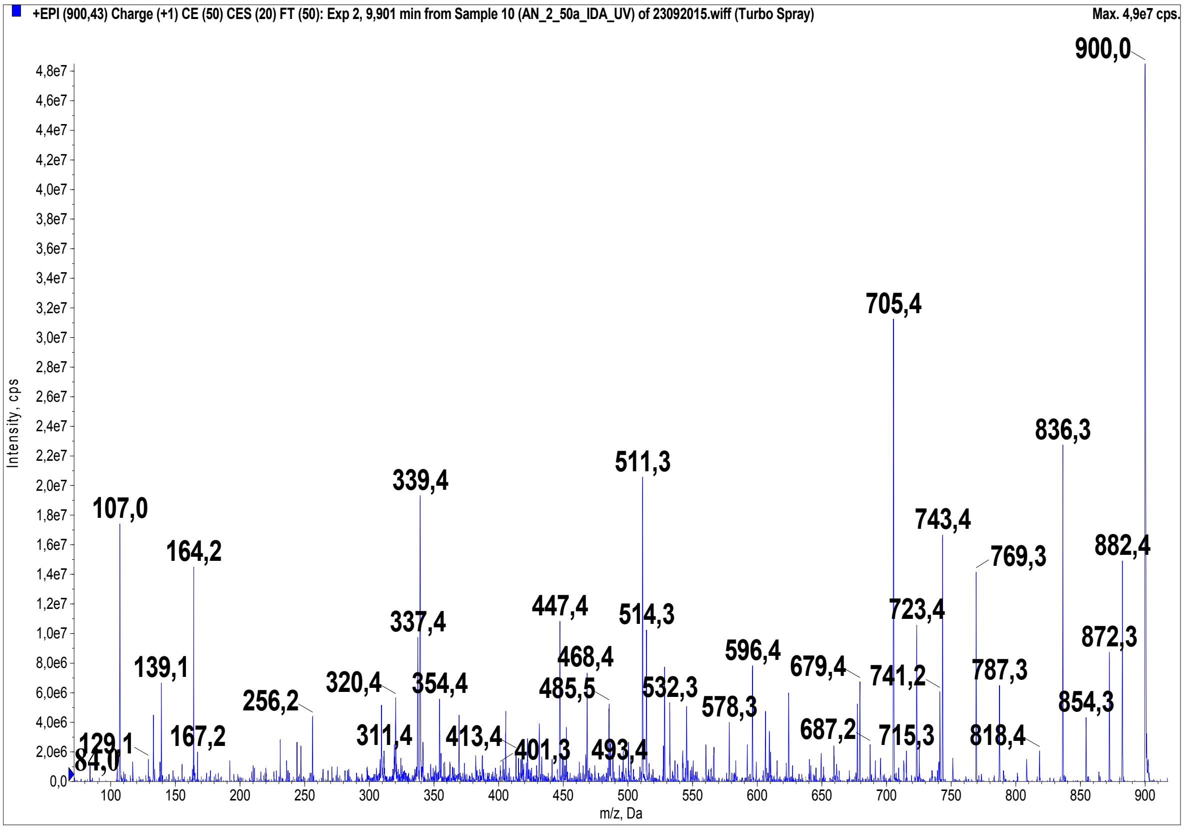


**Figure S3.** Mass fragmentation spectrum of anabaenopeptin with *m*/*z* of $[M + H]^+$ at 900 and structure Ile + CO + [Lys + Val + Hty + MeHty + MetO] elucidated on the basis of following fragments: 882 [M + H − $H_2O$], 872 [M + H − CO], 854 [M + H − $H_2O$ − CO], 836 [M + H − $CH_3SOH$ (from MetO)], 818 [M + H − $CH_3SOH$ − $H_2O$], 787 [M + H − Ile], 769 [M + H − Ile − $H_2O$], 743 [M + H − (CO + Ile)], 705 [M + H − Ile − $CH_3SOH$ − $H_2O$], 679 [M + H − (CO + Ile) − $CH_3SOH$], 528 [M + H − Ile − Hty − $CH_3SOH$ − $H_2O$], 511 [M + H − Ile − (Hty + Val)], 468 [MeHty + Hty + Val + H], 447 [M + H − Ile − (Hty + Val) − $CH_3SOH$], 339 [MetO + MeHty + H],164 MeHty, 107 [$CH_2PhOH$], 84 Lys–immonium ion.

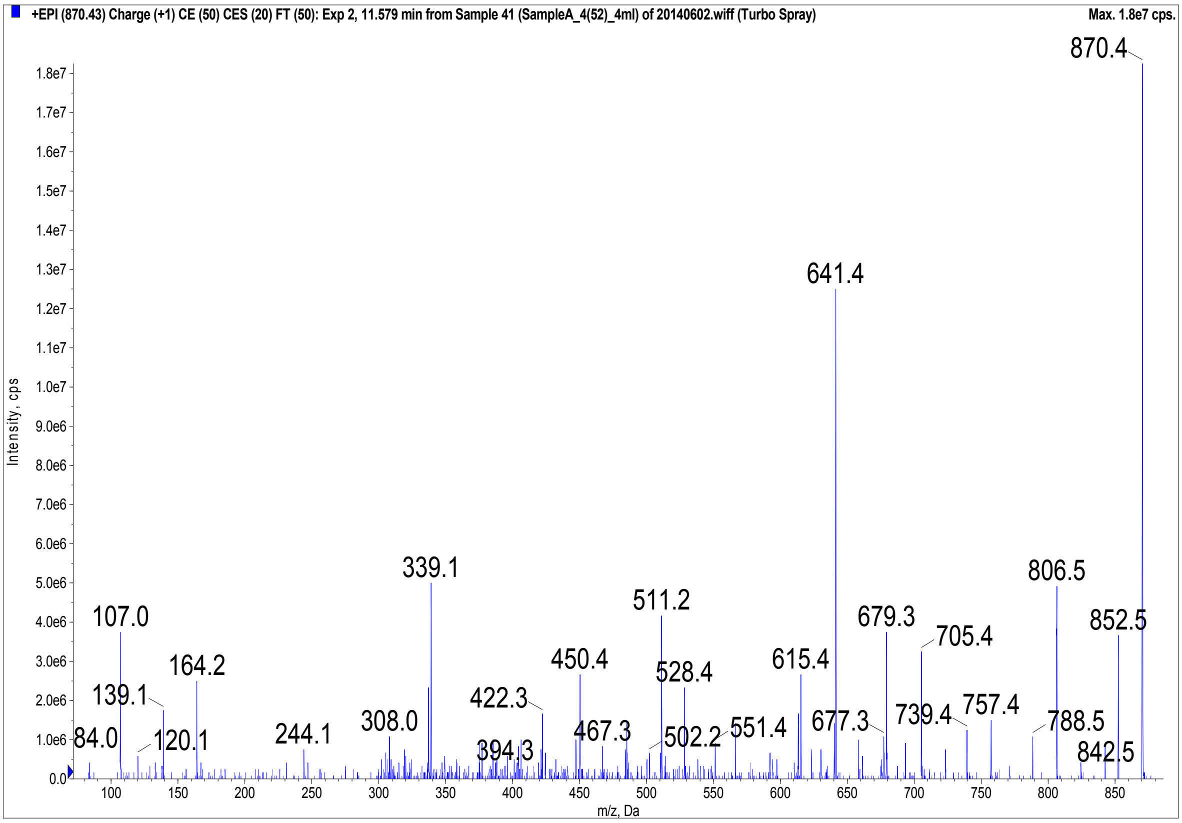


**Figure S4.** Mass fragmentation spectrum of anabaenopeptin with *m*/*z* of [M + H]$^+$ at 870 and structure [Phe + CO[Lys + Val + Leu + MeHty + MetO] elucidated on the basis of following fragments: 852[M + H − $H_2O$], 842 [M + H − CO], 806 [M + H − $CH_3SOH$ (from MetO)], 788 [M + H − $CH_3SOH$ − $H_2O$), 757 [M + H − Ile], 739 [M + H − Ile − $H_2O$], 705 [M + H − Phe − $H_2O$], 679 [M − MHty + H], 615 [M − Mhty + H − $CH_3SOH$], 641 [M + H − Phe − $CH_3SOH$ − $H_2O$], 528 [M + H − Phe − Leu − $CH_3SOH$ − $H_2O$], 511 [M + H − Phe − (Leu + Val)], 339 [MetO + MeHty + H], 164 MeHty, 120 Phe–immonium ion, 107 [$CH_2PhOH$], 84 Lys–immonium ion.

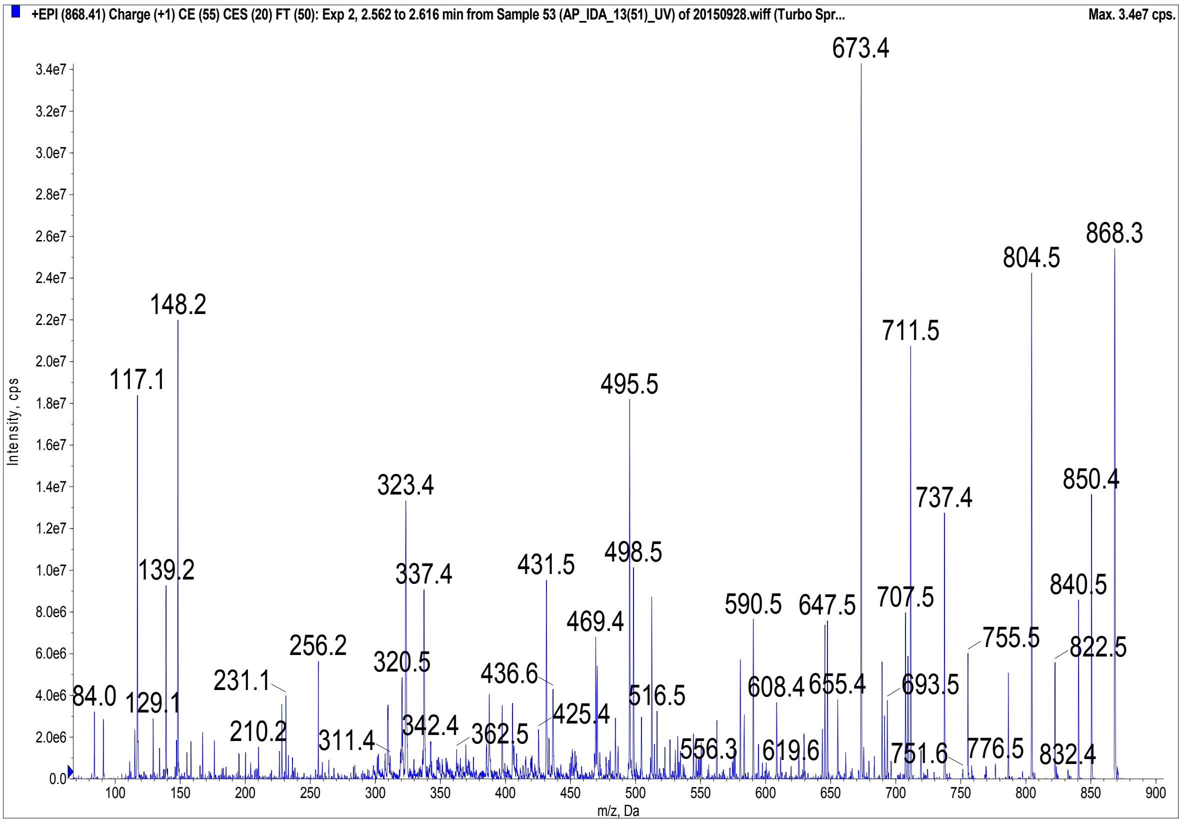


**Figure S5.** Mass fragmentation spectrum of anabaenopeptin with *m*/*z* of $[M + H]^+$ at 868 and structure Ile + CO[Lys + Val + Hph + MeHty + Met] elucidated on the basis of following fragments: 850[M + H − $H_2O$], 840 [M + H − CO], 822 [M + H − CO − $H_2O$], 755 [M + H − Ile], 737 [M + H − Ile − $H_2O$], 711 [M + H − (Ile + CO)], 707 [M + H − Hph], 693 [M + H − (Ile + CO) − $H_2O$], 608 [M + H − (Hph + Val)], 590 [M + H − (Hph + Val) − $H_2O$], 580 [M + H − (Hph + Val) − CO], 495 [M + H − Ile − (Hph + Val)], 323 [MeHty + Met + H], 84 Lys–immonium ion.

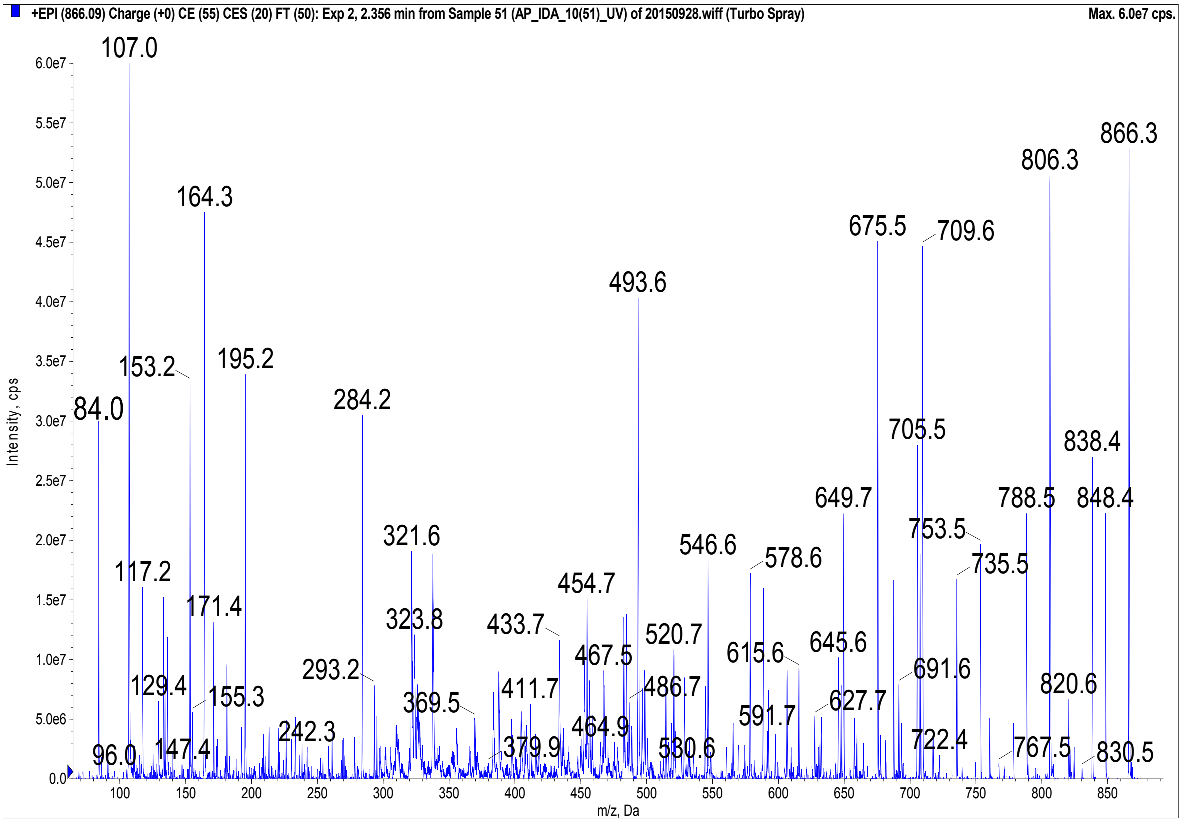


**Figure S6.** Mass fragmentation spectrum of anabaenopeptin with *m*/*z* of [M + H]$^+$ at **866** and structure Ile + CO[Lys + Val + Hph + MeHty + AcSer] elucidated on the basis of following fragments: 848 [M + H − $H_2O$], 838 [M + H − CO], 820 [M + H − CO − $H_2O$], 806 [M + H − $CH_3COOH$ (from AcSer)], 753 [M + H − Ile], 735 [M + H − Ile − $H_2O$], 675 [M + H − Ile − $CH_3COOH$ − $H_2O$], 709 [M + H − (CO + Ile)], 705 [M + H − Hph], 675 [M + H − MeHty], 649 [M + H − (CO + Ile) − $CH_3COOH$], 493 [M + H − Ile − (Hph + Val)], 337 [M + H − Ile − (Hph + MeHty) − $CH_3COOH$], 321 [MeHty + AcSer + H],164 MeHty, 107 [$CH_2PhOH$], 84 Lys–immonium ion.

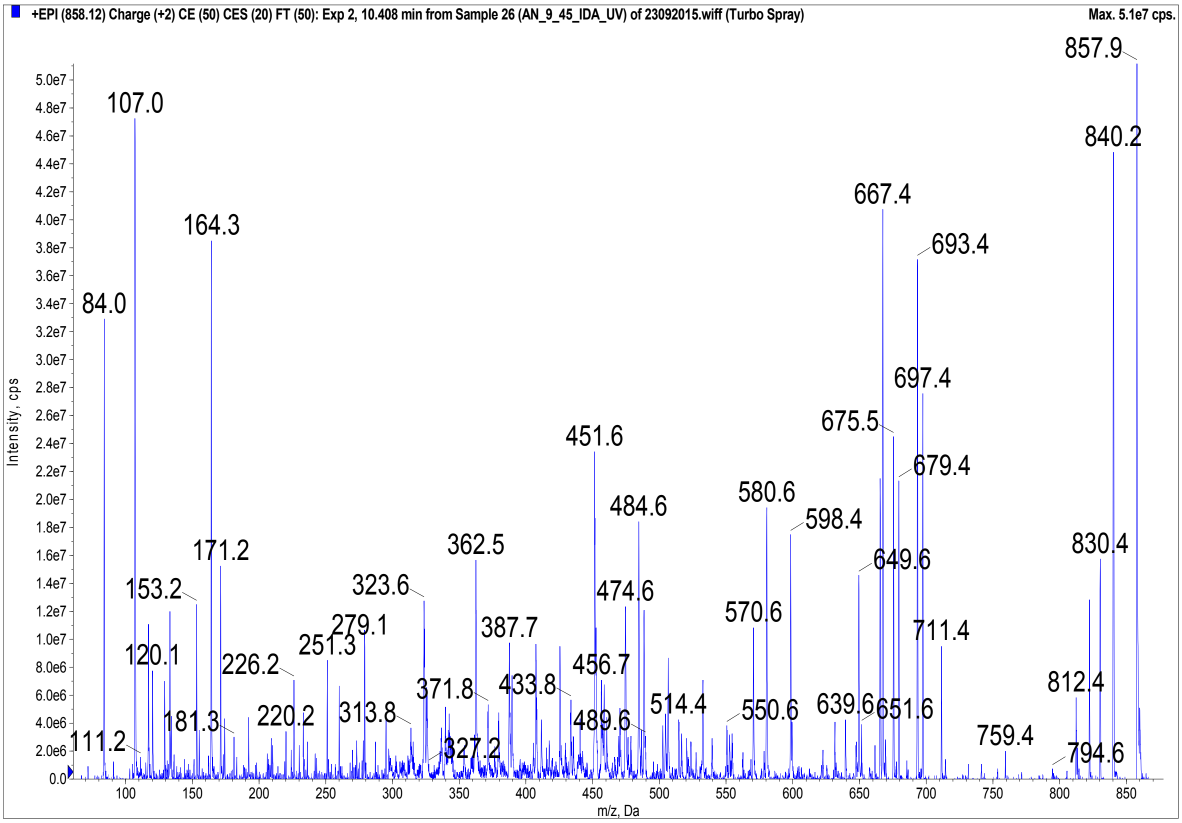


**Figure S7.** Mass fragmentation spectrum of anabaenopeptin with *m/z* of $[M + H]^+$ at **858** and structure Phe + CO[Lys + Val + Hph + MeHty + Ser] elucidated on the basis of following fragments: 840 [M + H − $H_2O$], 830 [M + H − CO], 812 [M + H − CO − $H_2O$], 711 [M + H − Phe], 697 [M + H − Hph], 667 [M + H − MeHty] and [M + H − (CO + Phe)], 649 [M + H − MeHty − $H_2O$], 639 [M + H − MeHty − CO], 598 [M + H − (Hph + Val)], 580 [M + H − (Hph + Val) − $H_2O$], 570 [M + H − (Hph + Val) − $H_2O$], 451 [M + H − Phe − (Hph + Val)], 279 [MeHty + Ser + H], 164 MeHty, 120 Phe–immonium ion, 107 [$CH_2PhOH$], 84 Lys–immonium ion.

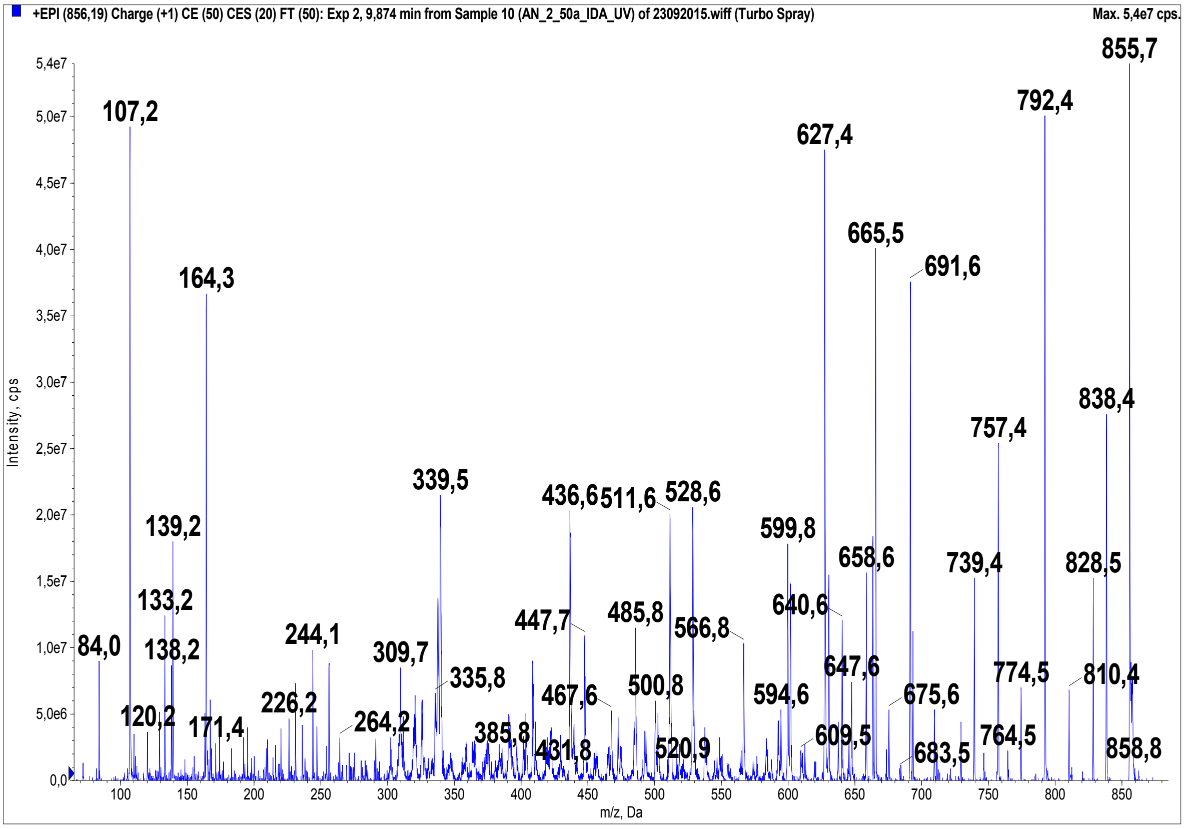


**Figure S8.** Mass fragmentation spectrum of anabaenopeptin with *m*/*z* of $[M + H]^+$ at 856 and structure Phe + CO + [Lys + Val + Val + MeHty + MetO] elucidated on the basis of following fragments 838 [M + H − $H_2O$], 828 [M + H − CO], 810 [M + H − CO − $H_2O$], 792 [M + H − $CH_3SOH$ (from MetO)], 757 [M + H − Val], 739 [M + H − Val − $H_2O$], 691 [M + H − Phe − $H_2O$], 658 [M + H − (Val + Val)], 647 [M + H − MeHty − $H_2O$], 627 [M + H − Phe − $CH_3SOH$ − $H_2O$], 566 [M + H − (Val + MeHty], 511 [M + H − Phe − (Val + Val)], 658 [M + H − (Val + Val], 447 [M + H − Phe − (Val + Val) − $CH_3SOH$], 339 [MetO − MeHty + H],164 MeHty, 120 Phe–immonium ion, 107 [$CH_2PhOH$], 84 Lys–immonium ion.

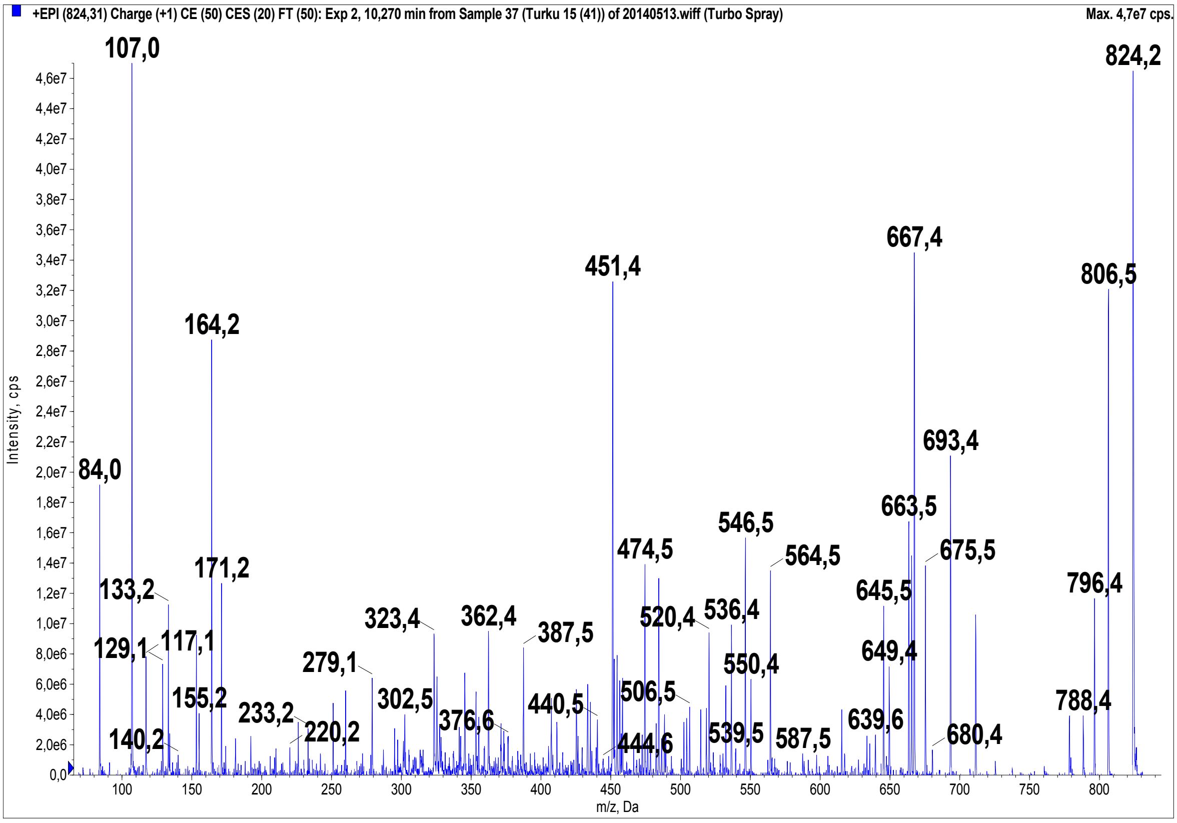


**Figure S9.** Mass fragmentation spectrum of anabaenopeptin with *m*/*z* of $[M + H]^+$ at 824 and structure Ile + CO + [Lys + Val + Hph + MeHty + Ser] elucidated on the basis of following fragments 806 [M + H − $H_2O$], 796 [M + H − CO], 778 [M + H − CO − $H_2O$], 711 [M + H − Ile], 693 [M + H − Ile − $H_2O$], 667 [M + H − (CO + Ile)], 663 [M + H − Hph], 645 [M + H − Hph − $H_2O$], 615 [M + H − MeHty − $H_2O$], 564 [M + H − (Hph + Val)], 546 [Hph + Val + Lys + CO + Ile + H], 520 [M + H − Ile − MeHty], 451 [M + H − (MeHty + Val) − Ile], 353 [Hph + MeHty + H], 279 [MeHty + Ser + H], 260 [MeHty + Ser + H − $H_2O$], 164 MeHty, 107 [$CH_2PhOH$], 84 Lys–immonium ion.

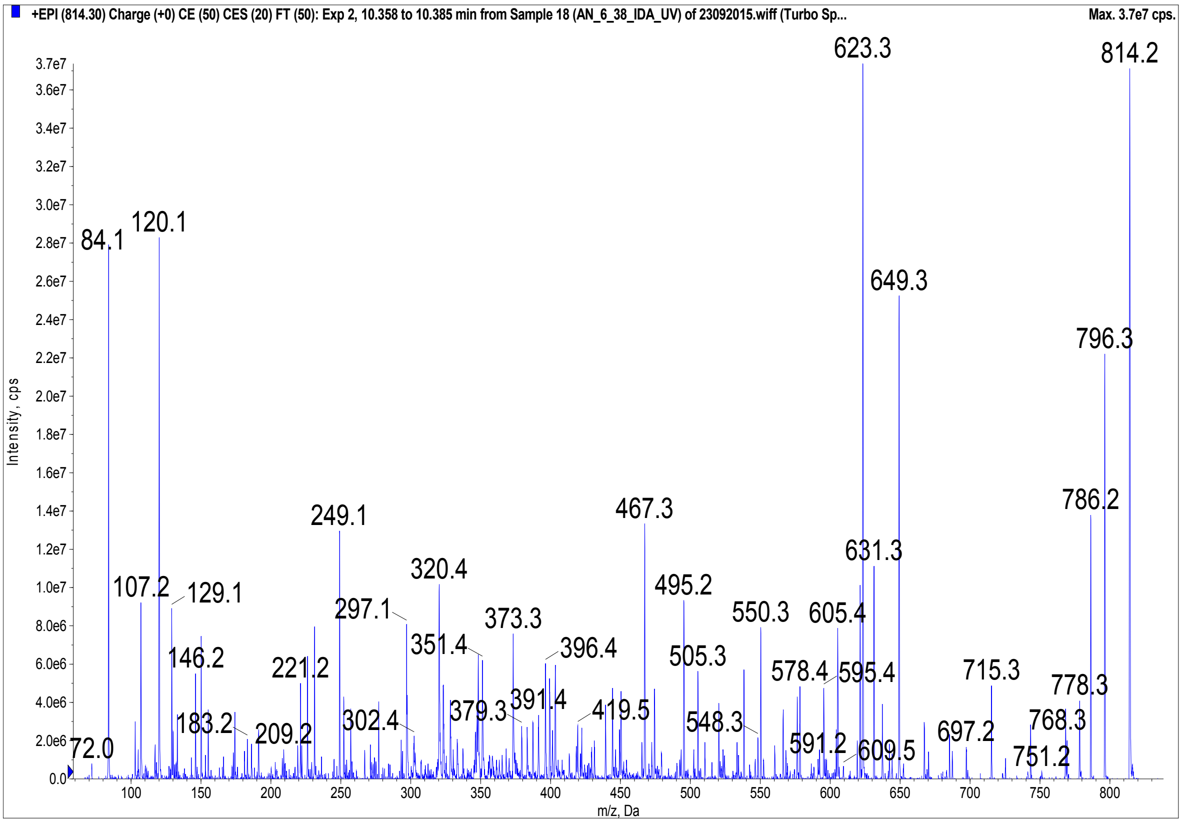


**Figure S10.** Mass fragmentation spectrum of anabaenopeptin with *m*/*z* of $[M + H]^+$ at **814** and structure Phe + CO[Lys + Val + Hty + MeGly + Phe] elucidated on the basis of following fragments: 796 [M + H − $H_2O$], 786 [M + H − CO], 768 [M + H − CO − $H_2O$], 715 [M + H − Val], 649 [M + H − Phe − $H_2O$], 623 [M + H − (CO + Phe)], 605 [M + H − (CO + Phe) − $H_2O$], 495 [Phe + MeGly + Hty + Val + H], 467 [Phe + Lys + CO + Phe + H], 396 [Hty + MeGly + Phe + H], 373 [M + H − Phe − (Hty + Val) − $H_2O$ ], 320 [M + H − Phe − (Val + Hty + MeGly + Phe)], 277 [Hty + Val + H], 249 [Hty + MeGly + H], 120 Phe immonium ion, 107 [$CH_2PhOH$], 84 Lys–immonium ion.

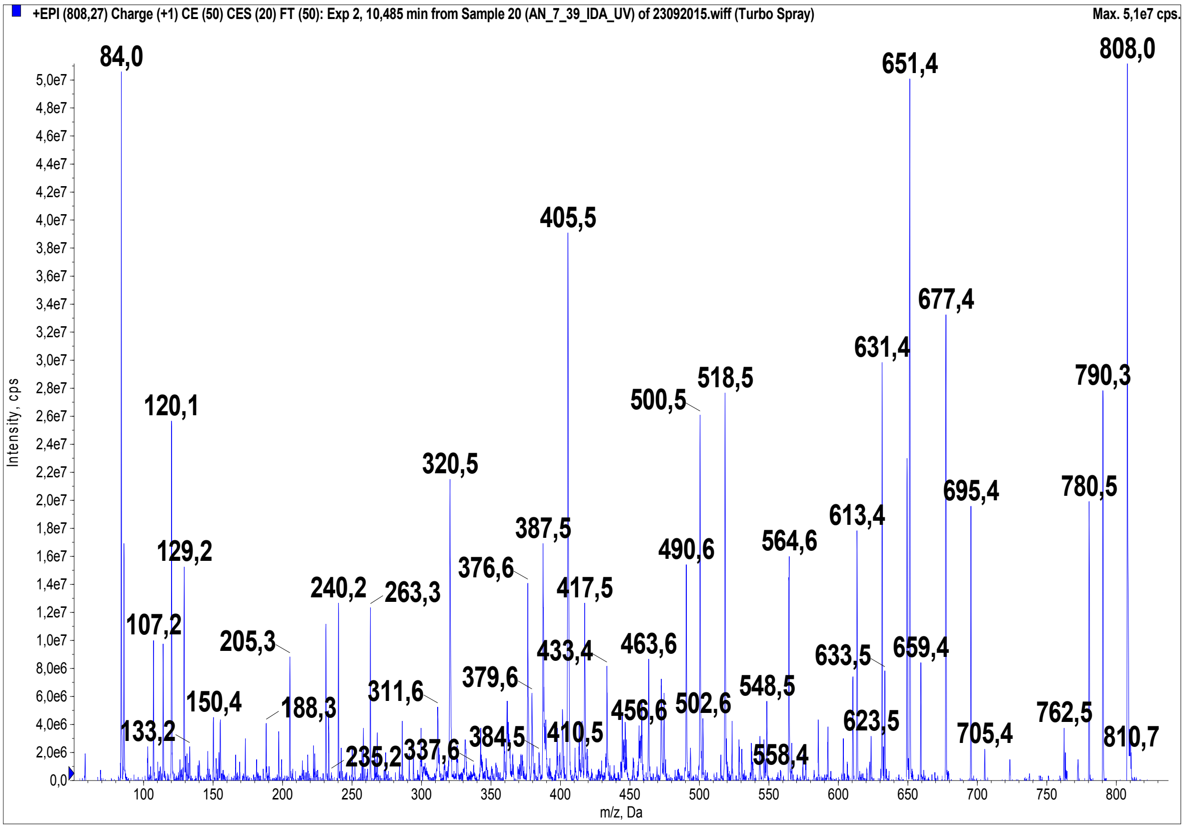


**Figure S11.** Mass fragmentation spectrum of anabaenopeptin with *m*/*z* of $[M + H]^+$ at 808 and structure Ile + CO + [Lys + Ile + Hty + MeAla + Phe] elucidated on the basis of following fragments: 790 [M + H − $H_2O$], 780 [M + H − CO], 762 [M + H − CO − $H_2O$], 695 [M + H − Ile], 677 [M + H − Ile − $H_2O$], 651 [M + H − (CO + Ile)], 631 [M + H − Hty], 613 [M + H − Hty − $H_2O$], 518 [M + H − Ile − Hty], 500 [M + H − Ile − Hty − $H_2O$], 490 [M + H − Ile − Hty − CO], 463 [M + H − (MeAla + Phe)], 405 [M + H − (Hty + $Ile^3$) − $Ile^1$], 387 [M + H − (Hty + $Ile^3$) − $Ile^1$ − $H_2O$], 320 [M + H − (MeAla + HTy + $Ile^3$) − $Ile^1$], 263 [MeAla + Hty + H], 120 Phe–immonium ion, 84 Lys–immonium ion.

**Table S1.** Published anabaenopeptin variants.

| | *m*/*z* | $AA_1$ (exo) | Ureido Linkage | $AA_2$ (D if Not Otherwise Indicated) | $AA_3$ | $AA_4$ | $AA_5$ | $AA_6$ | Source | Activity | Reference |
|---|---|---|---|---|---|---|---|---|---|---|---|
| **Arg in exocyclic position 1** | | | | | | | | | | | |
| AP 820, [Hph[4]] AP F | 821 | Arg | CO | Lys | Val | Hph | *N*MeAla | Phe | *Microcystis* sp. | Not determined | Welker *et al.* 2006, *Peptides* 27, 2090–2103. |
| Anabaenopeptin B | 837 | Arg | CO | Lys | Val | Hty | *N*MeAla | Phe | *Anabaena flos-aquae* NRC 525-17<br>*Anabaena*, Strain 90<br>*Oscillatoria agardii* NIES-204<br>AP B<br>*Planktothrix rubescence* bloom | rNEc, no PP1i<br>TRYi<br>No protease inh.<br>PP1i<br>ELAi, no TRY, no CHY, cytotoxicity<br>No CPAi | Harada 1995 *Tetraheddon Letters* 36, 1511–1514.<br>Repka *et al.* 2004 *Appl. Env. Microbiol.* 70, 4551–4560.<br>Murakami *et al.* 1997, *Phytochemistry* 44, 449–452<br>Gkelis *et al.* 2006, *Aquatic toxicology* 78, 32–41.<br>Bubik *et al.* 2008, *Biol. Chem.* 389, 1339–1346.<br>Murakami *et al.* 2000, *J. Nat. Prod.* 63, (9) 1280–1282. |
| Anabaenopeptin B1 | 851 | HArg | CO | Lys | Val | Hty | *N*MeAla | Phe | *P. rubescence* | Not determined | Ferranti *et al.* 2011, *Rapid Commun. Mass Spectrom.* 25, 1173–1183. |

| | | | | | | | | | | | |
|---|---|---|---|---|---|---|---|---|---|---|---|
| AP MM850 | 851 | Arg(OMe) | CO | Lys | Val | Hty | *N*MeAla | Phe | *Microcystis* sp. bloom | CHYi, ELAi, TRYi, no THROi | Zafir-Ilan and Carmeli 2010, *Tetrahedron* 66, 9194–9202. |
| Anabaenopeptin E | 851 | Arg | CO | Lys | Val | MeHty | *N*MeAla | Phe | *Oscillatoria agardhii* NIES-204 *Planktothrix agardhii* HUB 011 | Not determined Not determined no CPAi | Shin *et al*. 1997, *J. Nat. Prod*. 60, 139–141. Erhard *et al*. 1999, *Rapid Commun. Mass Spectrom*. 13, 337–343. Murakami *et al*. 2000, *J. Nat. Prod*. 63, (9) 1280–1282. |
| Anabaenopeptin F, APDA850 | 851 | Arg | CO | Lys | Ile/*allo*- | Hty | *N*MeAla | Phe | *Oscillatoria agardhii* NIES-204 *Planktothrix rubescens* NIES-610 *Oscillatoria agardhii* Strain 97 *P. rubescence*, bloom *Microcystis aeruginosa*, bloom | Not determined PP1i, PP2i Not determined ELAi, no THROi, no CHYi, cytotoxicity No TRYi, no THROi | Shin *et al*. 1997, *J. Nat. Prod*. 60, 139–141. Sano *et al*. 2001, *J. Nat. Prod*. 64, 1052–1055. Fujii etal. 2000, *Tetrahedron* 56, 725–733. Bubik *et al*. 2008, *Biol. Chem. 389,* 1339–1346. Adiv *et al*. 2013, *J. Nat. Prod*. 76, 2307–2315. |
| Anabaenopeptin F1, AP KT864 | 865 | HArg | CO | Lys | Ile | Hty | NMeAla | Phe | *Planktothrix rubescence* *Microcystis* sp. MB-K | Not determined No TRYi, no CHYi | Ferranti *et al*. 2011, *Rapid Commun. Mass Spectrom*. 25, 1173–1183. Beresovsky *et al*. 2006, *Isr. J. Chem* 46, 79–87. |

| | | | | | | | | | | | |
|---|---|---|---|---|---|---|---|---|---|---|---|
| Oscillamide B | 869 | Arg | CO | Lys | Met | Hty | NMeAla | Phe | *P. agardhii* CCAP 1459/11A | PP1i, PP2i | Sano *et al*. 2001, *J. Nat Prod*. 64, 1052–1055. |
| AP 877 | 878 | Arg | CO | Lys | Ile | Hph | *N*MeAla | Phe | *Desmonostoc* sp. | Not determined | Sanz *et al*. 2015, *Marine Drugs* 13, 3892–3919 |
| AP HU892 | 893 | Arg | CO | Lys | Val | Hph | NMeHty | Ile | *Microcystis aeruginosa* bloom | Not determined | Gesner-Apter and Carmeli 2009, *J. Nat. Prod*. 72, 1429–1436. |
| AP KB906 | 907 | Arg | CO | Lys | Ile | Hph | NMeHty | Ile | *Microcystis* spp. bloom | No TRYi, no CHYi | Elkobi-Peer and Carmeli 2015 *Marine Drugs* 13, 2347–2375. |
| AP 908 | 909 | Arg | CO | Lys | Val | Hty | *N*MeHty | Ile | *P. agardhii*, CYA 126/8 | CPAi, no TRYi no CHYi | Okumura *et al*. 2009, *J. Nat. Prod*. 72, 172–176. |
| Anabaenopeptin H | 923 | Arg | CO | Lys | Ile | Hty | NMeHty | Ile | *Oscillatoria agardhii* NIES-595 | CPAi | Itou *et al*. 1999, *Bioorganic & Medical Chem. Lett*. 9, 1243–1246. |
| Oscillamide C | 957 | Arg | CO | Lys | Ile | Hty | NMeHty | Phe | *P. rubescens* CCAP 1459/14 | PP1i, PP2i | Sano *et al*. 2001, *J. Nat Prod*. 64, 1052–1055. |
| AP 891 | 892 | Arg | CO | Lys | Ile | MeHph | *N*MeAsn | Phe | *Desmonostoc* sp. | Not determined | Sanz *et al*. 2015, *Marine Drugs* 13, 3892–3919. |
| AP 905 | 906 | Arg | CO | Lys | Ile | EtHph | *N*MeAsn | Phe | *Desmonostoc* sp. | Not determined | Sanz *et al*. 2015, *Marine Drugs* 13, 3892–3919. |
| Paltolide A | 812 | Arg | CO | Lys | Ala | Leu | Leu | Trp | *Theonella swinhoei* | No cytotoxicity | Plaza *et al*. 2010, *J. Nat. Prod*. 73, 485–488. |
| Paltolide B | 842 | Arg | CO | Lys | Ala | Leu | *N*MeLeu | L-5'-hydroxyTrp | *Theonella swinhoei* | No cytotoxicity | Plaza *et al*. 2010, *J. Nat. Prod*. 73, 485–488. |

| | | | | | | | | | | | |
|---|---|---|---|---|---|---|---|---|---|---|---|
| Paltolide C | 904 | Arg | CO | Lys | Ala | Leu | *N*MeLeu | L-6'-BrTrp | *Theonella swinhoei* | No cytotxicity | Plaza *et al*. 2010, *J. Nat. Prod.* 73, 485–488. |
| AP 906 (putative) | 907 | Arg | CO | Lys | Ile | MeHty | *N*MeLeu | Phe | *Microcystis* sp. | Not determined | Puddick *et al*. 2008, *Chemistry in New Zealand* 72, 25–28. |
| Anabaenopeptin G | 909 | Arg | CO | Lys | Ile | Hty | *N*MeLeu | Tyr | *Planktothrix agardhii* HUB 011 | Not determined | Erhard *et al*. 1999, *Rapid Commun. Mass Spectrom.* 13, 337–343. |
| **Glu(OMe) in exocyclic position** | | | | | | | | | | | |
| AP MM822 | 823 | Glu(OMe) | CO | Lys | Val | Hty | *N*MeAla | Phe | *Microcystis* sp. | CHYi, ELAi, no THROi, no TRYi | Zafir-Ilan and Carmeli 2010, *Tetrahedron* 66, 9194–9202. |
| **Ile in exocyclic position 1** | | | | | | | | | | | |
| Anabenopeptin I | 760 | Ile | CO | Lys | Val | Hty | *N*MeAla | Leu | *Aphanizomenon flos-aquae* NIES-81 | CPAi | Murakami *et al*. 2000, *J. Nat. Prod.* 63, 1280–1282. |
| Scizopeptin 791 | 792 | Ile | CO | Lys | Ile | Hph | *N*MeAla | Phe | Terrestial *Schizothrix* sp. | TRYi, no CHYi | Reshef and Carmeli 2002, *J. Natural Products* 65, 1187–1189. |
| Anabenopeptin J | 794 | Ile | CO | Lys | Val | Hty | *N*MeAla | Phe | *Aphanizomenon flos-aquae* NIES-81 | CPAi | Murakami *et al*. 2000, *J. Nat. Prod.* 63, 1280–1282. |
| AP 807 | 808 | Ile | CO | Lys | Ile | Hty | *N*MeAla | Phe | *Nodularia spumigena*, Australian strains Baltic Sea bloom | Not determined CPAi, PP1i | Mazur-Marzec *et al*. 2013, *Marine Drugs* 11, 1–19. This study |
| NP 823 | 824 | Ile | CO | Lys | Val | Hph | *N*MeHty | Ser | Baltic Sea bloom | Not determined | This study |
| Nodulapeptin 855 | 856 | Ile | CO | Lys | Met | Hph | *N*MeHty | Ser | *Nodularia spumigena* CCNP1402 | Not determined | Mazur-Marzec *et al*. 2013, *Marine Drugs* 11, 1–19. |

| | | | | | | | | | | | |
|---|---|---|---|---|---|---|---|---|---|---|---|
| Anabaenopeptin T | 866 | Ile | CO | Lys | Val | Hty | *N*MeHty | Ile | Bloom material, lake Teganuma, Japan | CPAi | Kodani *et al.* 1999, *FEMS Microbiol. Lett.* 178, 343–348. |
| NP 865 | 866 | Ile | CO | Lys | Val | Hph | *N*MeHty | AcSer | Baltic sea bloom | CPAi, PP1i | This study |
| NP 867 | 868 | Ile | CO | Lys | Val | Hph | *N*MeHty | Met | Baltic sea bloom | CPAi, PP1i | This study |
| NP 879 | 880 | Ile | CO | Lys | Ile | Hph | *N*MeHty | AcSer | *Nodularia spumigena* CCNP 1402, BY1,Node2, Nodg3, Nodh2 | Not determined | Mazur-Marzec *et al.* 2013, *Marine Drugs* 11, 1–19. |
| Nodulapeptin 881 | 882 | Ile | CO | Lys | Ile | Hph | *N*MeHty | Met | *Nodularia spumigena* CCNP 1402 | Not determined | Mazur-Marzec *et al.* 2013, *Marine Drugs* 11, 1–19. |
| NP 883 | 884 | Ile | CO | Lys | Val | Hph | *N*MeHty | Met(O) | Baltic sea bloom | CPAi, PP1i | This study |
| Nodulapeptin C | 898 | Ile | CO | Lys | Met | Hph | *N*MeHty | AcSer | *Nodularia spumigena* CCY9414 | Not determined | Rouhiainen *et al.* 2010, *Chemistry & Biology* 17, 265–273. |
| NP 899 | 900 | Ile | CO | Lys | Val | Hty | *N*MeHty | Me(O) | Baltic Sea bloom | PP1i, CPAi | This study |
| [Met][6] Nodulapeptin C | 900 | Ile | CO | Lys | Met | Hph | *N*MeHty | Met | *Nodularia spumigena* CCY9414 | Not determined | Rouhiainen *et al.* 2010, *Chemistry & Biology* 17, 265–273. |
| Nodulapeptin B | 914 | Ile | CO | Lys | Met(O) | Hph | *N*MeHty | AcSer | *Nodularia spumigena* AV1 | Not determined | Fujii *et al.* 1997, *Tetrahedron Letters* 31, 5525–5528. |
| NP 915 | 916 | Ile | CO | Lys | Ile | Hph | *N*MeHty | Met | *Nodularia spumigena* , CCNP 1402, BY1, Node2, Nodg3, Nodh2 | Not determined | Mazur-Marzec *et al.* 2013, *Marine Drugs* 11, 1–19. |
| Nodulapeptin A | 930 | Ile | CO | Lys | Met($O_2$) | Hph | *N*MeHty | AcSer | *Nodularia spumigena* AV1 | Not determined | Fujii *et al.* 1997, *Tetrahedron Letters* 31, 5525–5528. |
| NP931 | 932 | Ile | CO | Lys | Met(O) | Hph | *N*MeHty | Met(O) | *Nodularia spumigena* CCNP1402, BY1 | Not determined | Mazur-Marzec *et al.* 2013, *Marine Drugs* 11, 1–19. |
| Nodulapeptin 855 | 856 | Ile | CO | Lys | MetO | Hph | *N*MeHph | Ser | *Nodularia spumigena* Baltic Sea and turkish strains | Not determined | Mazur-Marzec *et al.* 2013, *Marine Drugs* 11, 1–19. |

| | | | | | | | | | | | |
|---|---|---|---|---|---|---|---|---|---|---|---|
| [Ser][6] Nodulapeptin B | 872 | Ile | CO | Lys | MetO | Hph | *N*MeHph | Ser | *Nodularia spumigena* CCY9414 | Not determined | Rouhiainen *et al.* 2010, *Chemistry & Biology* 17, 265–273. |
| Nodulapeptin 881 | 882 | Ile | CO | Lys | Met | Hph | *N*MeHph | AcSer | *Nodularia spumigena* CCNP 1402, BY1 | Not determined | Mazur-Marzec *et al.* 2013, *Mar. Drugs* 11, 1–19 |
| Nodulapeptin 883 | 884 | Ile | CO | Lys | MetO | Hph | *N*MeHph | Met | *Nodularia spumigena* CCNP 1402 | Not determined | Mazur-Marzec *et al.* 2013, *Mar. Drugs* 11, 1–19. |
| [MHph][5] Nodulapeptin B | 898 | Ile | CO | Lys | MetO | Hph | *N*MeHph | AcSer | *Nodularia spumigena* CCY9414 | not determined | Rouhiainen *et al.* 2010, *Chemistry & Biology* 17, 265–273. |
| Brunsvicamide A | 845 | Ile | CO | L-Lys<br>D-Lys | Val | Leu | *N*Me-L-5'-hydroxyTrp | Phe | *Tychonema* | PPi (MptpB, weak)<br>No PPi, CPAi and CPBi | Müller *et al.* 2006, *J. Med. Chem.* 49, 4871–4878.<br>Walther *et al.* 2009, *ChemBioChem* 10, 1153–1162. |
| Brunsvicamide B | 859 | Ile/*allo*-Ile | CO | L-Lys | Ile | Leu | *N*Me-L-5'-hydroxyTrp | Phe | *Tychonema* | PPi (MptpB) | Muller *et al.* 2006, *J. Med Chem.* 49, 4871–4878. |
| Mozamide A | 861 | L-*allo*-Ile | CO | L-Lys | D-Val | Leu | *N*Me-L-5'-hydroxyTrp | Phe | *Theonella*, sponge | No anti-microbial activity | Schmidt *et al.* 1997, *J. Nat. Prod* 60, 779–782. |
| Mozamide B | 875 | L-*allo*-Ile | CO | L-Lys | D-Ile | Leu | *N*Me-L-5'-hydroxyTrp | Phe | *Theonella*, sponge | No anti-microbial activity | Schmidt *et al.* 1997, *J. Nat. Prod* 60, 779–782. |
| Brunsvicamide C | 877 | Ile/*allo*-Ile | CO | L-Lys | Val | Leu | *N*Me-L-*N*'-formyl-D-kynurenine | Phe | *Tychonema* | PPi (MptpB) | Muller *et al.* 2006, *J. Med Chem.* 49, 4871–4878. |
| (−)-Psymbamide | 937 | Ile | CO | Lys | Leu | Leu | *N*Me-L-5'-BrTrp | Phe | Sponge *Psammocinia* aff. *bulbosa* | Not determined | Robinson *et al.* 2007, *J. Nat. Prod.* 70, 1002–1009. |
| Pompanopeptin B | 958 | Ile | CO | Lys | Val | Hty | *N*MeAhpha | Htyr | *Lyngbya confervoides*, bloom | Not determined | Matthew *et al.* 2008, *Tetrahedron* 64, 4081–4089. |

| | | | | | | | | | | | |
|---|---|---|---|---|---|---|---|---|---|---|---|
| **Leu in exocyclic position 1** | | | | | | | | | | | |
| AP 848 | 849 | Leu | CO | Lys | Ile | MeHph | *N*MeAsn | Phe | *Desmonostoc* sp. | Not determined | Sanz *et al*. 2015, *Marine Drugs* 13, 3892–3919. |
| AP 862 | 863 | Leu | CO | Lys | Ile | EtHph | *N*MeAsn | Phe | *Desmonostoc* sp. | Not determined | Sanz *et al*. 2015, *Marine Drugs 13, 3892–3919.* |
| Konbamide | 877 | Leu | CO | L-Lys | Ala | Leu | *N*MeLeu | BhTrp | *Theonella*, sponge | CAM-PDE inhibition | Kobayashi *et al.* 1991, *J. Chem. Soc. Chem. Commun*. 1050–1052 Schmidt and Weinbrenner 1996, *Angew. Chem. Int. Ed. Engl*. 35(12) 1336–1338 |
| **Lys in exocyclic position 1** | | | | | | | | | | | |
| Anabaenopeptin C | 809 | Lys | CO | Lys | Val | Hty | *N*MeAla | Phe | *Anabaena* sp. 90 | Not determined | Fujii *et al*. 1996, In *Harmful and Toxic Algal Blooms* 559–562. |
| AP 849 | 850 | Lys | CO | Lys | Ile | Hph | *N*MeAsn | Phe | *Desmonostoc* sp. | Not determined | Sanz *et al*. 2015, *Marine Drugs* 13, 3892–3919. |
| AP 863 | 864 | Lys | CO | Lys | Ile | MeHph | *N*MeAsn | Phe | *Desmonostoc* sp. | Not determined | Sanz *et al*. 2015, *Marine Drugs* 13, 3892–3919. |
| AP 877 | 878 | Lys | CO | Lys | Ile | EtHph | *N*MeAsn | Phe | *Desmonostoc* sp. | Not determined | Sanz *et al*. 2015, *Marine Drugs* 13, 3892–3919. |
| **Phe in exocyclic position 1** | | | | | | | | | | | |
| AP 813 | 814 | Phe | CO | Lys | Val | Hty | *N*MeGly | Phe | Baltic Sea bloom | PP1i, CPAi | This study |

| | | | | | | | | | | | |
|---|---|---|---|---|---|---|---|---|---|---|---|
| Anabaenopeptin D | 828 | Phe | CO | Lys | Val | Hty | *N*MeAla | Phe | *Anabaena* sp. 202 A2/41 (*A. lemmermannii*), Baltic Sea bloom | not determined PP1i, CPAi | Fujii *et al*. 1996, In *Harmful and Toxic Algal Blooms* 559–562. This study |
| AP 841 | 842 | Phe | CO | Lys | Ile | Hty | *N*MeAla | Phe | *Nodularia spumigena* CCNP 1401,1403, B15a | not determined | Mazur-Marzec *et al*. 2013, *Mar. Drugs* 11, 1–19 |
| AP 841 | 842 | Phe | CO | Lys | Val | Hph | *N*MeAla | Hty | *Desmonostoc* sp. | not determined | Sanz *et al*. 2015, *Marine Drugs* 13, 3892–3919. |
| Lyngbyaureidamide B | 842 | D-Phe | CO | Lys | Ile | Hty | *N*MeAla | Phe | *Lyngbya* sp. SAG 36.91 | No CHY-like inh. | Zi *et al*. 2012, *Phytochemistry* 74, 173–177. |
| Lyngbyaureidamide A | 856 | D-Phe | CO | Lys | Ile | Hty | *N*MeAla | Hph | *Lyngbya* sp. SAG 36.91 | No CHY-like inh. | Zi *et al*. 2012, *Phytochemistry* 74, 173–177. |
| AP 855 | 856 | Phe | CO | Lys | Ile | Hph | *N*MeAla | Hty | *Nostoc* sp. | Not determined | Sanz *et al*. 2015, *Marine Drugs* 13, 3892–3919. |
| AP 855 | 856 | Phe | CO | Lys | Val | Val | *N*MeHty | Met(O) | Baltic Sea bloom | PP1i, CPAi | This study |
| AP 857 | 858 | Phe | CO | Lys | Val | Hty | *N*MeAla | Hty | *Nostoc* sp. | Not determined | Sanz *et al*. 2015, *Marine Drugs* 13, 3892–3919. |
| AP 857 | 858 | Phe | CO | Lys | Val | Hph | *N*MeHty | Ser | Baltic Sea bloom | PP1i, CPAi | This study |
| NP 869 | 870 | Phe | CO | Lys | Val | Leu | *N*MeHty | Met(O) | Baltic Sea bloom | PP1i | This study |
| AP 871 | 872 | Phe | CO | Lys | Ile | Hty | *N*MeHty | Hty | *Nostoc* sp. | Not determined | Sanz *et al*. 2015, *Marine Drugs* 13, 3892–3919. |
| NP 899 | 900 | Phe | CO | Lys | Val | Hph | *N*MeHty | AcSer | *Nodularia spumigena* KAC 66 Baltic Sea bloom | Not determined PP1i, CPAi | Schumacher *et al.* 2012, *Tetrahedron* 68, 1622–1628. This study |
| NP 901 | 902 | Phe | CO | Lys | Val | Hph | *N*MeHty | Met | *Nodularia spumigena* KAC 66 | Not determined | Schumacher *et al.* 2012, *Tetrahedron* 68, 1622–1628. |

| | | | | | | | | | | | |
|---|---|---|---|---|---|---|---|---|---|---|---|
| NP 915 | 916 | Phe | CO | Lys | Val | Hty | *N*MeHty | AcSer | *Nodularia spumigena* KAC66, CCNP 1423, CCNP 1424, CCNP 1425 Baltic Sea bloom | PP1i, CPAi | Mazur-Marzec *et al.* 2013, *Marine Drugs* 11, 1–19. This study |
| NP 917 | 918 | Phe | CO | Lys | Val | Hph | *N*MeHty | Met(O) | *Nodularia spumigena* KAC 66 Baltic Sea bloom | Not determined PP1i, CPAi | Schumacher *et al.* 2012, *Tetrahedron* 68, 1622–1628. This study |
| NP 933 | 934 | Phe | CO | Lys | Val | Hty | *N*MeHty | Met(O) | *Nodularia spumigena* CCNP 1423, CCNP 1424, CCNP 1425 Baltic Sea bloom | PP1i, CPAi | Mazur-Marzec *et al.* 2013, *Marine Drugs* 11, 1–19. This study |
| NP 883 | 884 | Phe | CO | Lys | Val | Hph | NMeHph | AcSer | *Nodularia spumigena* KAC66, CCNP 1423, CCNP 1424, CCNP 1425 | not determined | Mazur-Marzec *et al.* 2013, *Marine Drugs* 11, 1–19. |
| AP 813 | 814 | Phe | CO | Lys | Val | Hty | *N*MeGly | Phe | Baltic Sea bloom | PP1i, CPAi | This study |
| AP NZ825 | 826 | Phe | CO | Lys | Ile | Hph | *N*MeGly | Hph | *Anabaena* sp. TAU strain NZ-3-1 | No activity towards serine proteaser | Grach-Progrebinsky and Carmeli 2008, *Tetrahedron* 64, 10233–10238. |
| AP NZ841 | 842 | Phe | CO | Lys | Ile | Hty | *N*MeGly | Hph | *Anabaena* sp. TAU strain NZ-3-1 | No activity towards serine proteaser | Grach-Progrebinsky and Carmeli 2008, *Tetrahedron* 64, 10233–10238. |
| Nostamide A | 842 | Phe | CO | Lys | Ile | Hph | *N*MeGly | Hty | *Nostoc punctiforme* PCC73102 | Not determined | Rouhiainen *et al.* 2010, *Chemistry & Biology* 17, 265–267. |
| AP NZ857 | 858 | Phe | CO | Lys | Ile | L-Hty | *N*MeGly | Hty | *Anabaena* sp. TAU strain NZ-3-1 *Nostoc punctiforme* PCC73102 | No activity towards serine proteaser | Grach-Progrebinsky and Carmeli 2008, *Tetrahedron* 64, 10233–10238. Rouhiainen *et al.* |

| | | | | | | | | | | | |
|---|---|---|---|---|---|---|---|---|---|---|---|
| | | | | | | | | | | | 2010, *Chemistry & Biology* 17, 265–273. |
| AP 882 | 883 | Phe | CO | Lys | Ile | MeHph | *N*MeAsn | Phe | *Nostoc* sp. | Not determined | Sanz *et al*. 2015, *Marine Drugs* 13, 3892–3919. |
| AP 896 | 897 | Phe | CO | Lys | Ile | EtHph | *N*MeAsn | Phe | *Nostoc* sp. | Not determined | Sanz *et al*. 2015, *Marine Drugs* 13, 3892–3919. |
| Keramamide A | 943 | Phe | CO | L-Lys | Leu | Leu | *N*MeCht | Phe | *Theonella*, sponge | No cytotoxicity, SERCA inhibition | Kobyashi *et al*. 1991, *J. Chem. Soc. Perin. Trans.* 1, 2609–2611. |
| Keramamide L | 927 | Phe | CO | L-Lys | Leu | Leu | *N*MeCTrp | Phe | *Theonella*, sponge | Cytotoxicity | Uemoto *et al*. 1999, *Tetrahedron* 55, 12543–12548. |
| **Trp in exocyclic position 1** | | | | | | | | | | | |
| Ferintoic acid A | 867 | Trp | CO | Lys | Val | Htyr | *N*MeAla | Phe | *Microcystis aeruginosa* bloom | No CHYi | Williams *et al*. 1996, *J. Nat. Prod.* 59, 570–575. |
| Ferintoic acid B | 881 | Trp | CO | Lys | *allo*-Ile | Htyr | *N*MeAla | Phe | *Microcystis aeruginosa* bloom | No CHYi | Williams *et al*. 1996, *J. Nat. Prod.* 59, 570–575. |
| **Tyr in exocyclic position 1** | | | | | | | | | | | |
| Anabaenopeptin A | 844 | Tyr | CO | Lys | Val | Hty | *N*MeAla | Phe | *Anabaena flos-aquae* NRC 525-17<br>Baltic Sea bloom | rNEc, no Ppi<br>PP1i<br>PP1i, CPAi | Harada *et al*. 1995, *Tetraheddon Letters* 36, 1511–1514.<br>Gkelis *et al*. 2006, *Aquatic Toxicology* 78, 32–41.<br>This study |
| Oscillamide Y | 858 | Tyr | CO | Lys | Ile | Hty | *N*MeAla | Phe | *Oscillatoria agardhii*= *P. rubescense* NIES-610<br>Synthetic and | CHYi<br>No CHYi<br>PP1i, CPAi | Sano and Kaya 1995, *Tetrahedron lett.* 36, 5933–5936.<br>Marsh *et al*. 1997, *J. Org. Chem.* 62, |

| | | | | | | | | | | | |
|---|---|---|---|---|---|---|---|---|---|---|---|
| | | | | | | | | | natural<br>Baltic Sea bloom | | 6199–6203.<br>This study |
| AP KB899 | 900 | Tyr | CO | Lys | Val | Hph | *N*MeHty | Ile | *Microcystis* spp. bloom | No TRYi, no CHYi | Elkobi-Peer and Carmeli 2015, *Mar. Drugs* 13, 2347–2375. |
| AP MM913 | 914 | Tyr | CO | Lys | Ile | Hph | *N*MeHty | Ile | *Microcystis* sp., bloom | No endoprotease inhibition | Zafir-Ilan and Carmeli 2010, *Tetrahedron* 66, 9194–9202. |
| AP 915 | 916 | Tyr | CO | Lys | Ile | Hty | *N*MeHty | Ile | *P. agardhii*, CYA 126/8 | No TRYi, no CHYi | Okumura *et al.* 2009, *J. Natural Products* 72, 172–176. |
| Anabaenopeptin G * | 930 | Tyr | CO | Lys | Ile | Hty | *N*MeHty | Ile | *Oscillatoria agardhii* NIES-595 | CPAi | Itou *et al.* 1999, *Bioorganic & Medical Chem. Lett.* 9, 1243–1246. |
| Oscillamide H | 930 | Tyr | CO | Lys | *N*MeIle | Ile | *N*MeHty | Ile | *Planktothrix agardhii*, NIES-595 | Not determined | Sano *et al.* 1996, *Tennen Yuki Kagobatsu Toronkai Koen Yoshishu* 38, 433–438. Dr T. Sano, personal communication |
| **Val in exocyclic position 1** | | | | | | | | | | | |
| AP 802 | 803 | Val | CO | Lys | Ile | Trp | *N*MeAla | Phe | *Brasilonema* spp. | Not determined | Sanz *et al.* 2015, *Marine Drugs* 13, 3892–3919. |

Abbreviations: BhTrp, 2-bromo-5-hydroxytryptophan. CAM-PDE, calmodulin -activated brain phosphodiesterase. CHY, chymotrypsin. CPA, carboxypeptidase A. ELA, elastase. i, inhibition. MeCht, 6-chloro-5-hydroxy-*N*-methyltryptophan. MptpB, *Mycobacterium tuberculosis* protein tyrosine phophatase B. PP, protein phosphatase. rNec, norepinephrin induced contraction. SERCA, sarcoplasmic reticulum $Ca^{2+}$-ATPase. TRY, trypsin. THRO, thrombin.
